# Mitochondrial oxygen tension in critically ill patients receiving red blood cell transfusions: a multicenter observational cohort study

**DOI:** 10.1186/s40635-024-00646-3

**Published:** 2024-07-08

**Authors:** M. Baysan, B. Hilderink, L. van Manen, C. Caram-Deelder, E. G. Mik, N. P. Juffermans, J. G. van der Bom, M. S. Arbous

**Affiliations:** 1 Department of Intensive Care Medicine, Leiden University Medical Center, Albinusdreef 2, 2333 ZA Leiden, The Netherlands; 2 Department of Clinical Epidemiology, Leiden University Medical Center, Leiden, The Netherlands; 3 Jon J Van Rood Center for Clinical Transfusion Research, Sanquin/LUMC, Leiden, The Netherlands; 4 Department of Intensive Care Medicine, Amsterdam UMC, Location AMC, Amsterdam, The Netherlands; 5 Department of Intensive Care Medicine, OLVG Hospital, Amsterdam, The Netherlands; 6 Department of Anesthesiology, Laboratory of Experimental Anesthesiology, Erasmus MC- University Medical Center Rotterdam, Rotterdam, The Netherlands; 7 Laboratory of Translational Intensive Care, Erasmus MC, Rotterdam, The Netherlands

**Keywords:** Anemia, Red blood cell transfusion, Tissue oxygenation, Mitochondrial oxygen tension

## Abstract

**Purpose:**

Currently, there is no marker of efficacy of red blood cell (RBC) transfusion. This study describes the impact of RBC transfusion on mitochondrial oxygen tension (mitoPO_2_) and mitochondrial oxygen consumption (mitoVO_2_) in critically ill patients with anemia.

**Methods:**

Critically ill patients with a hemoglobin concentration < 10 g/dL, for whom a single RBC unit had been ordered, were included. MitoPO_2_ was measured with the COMET device immediately before RBC transfusion, 0.5 h, 1 h, 3 h, and 24 h after RBC transfusion. MitoVO_2_ was calculated from dynamic mitoPO_2_ measurements during cessation of local oxygen supply.

**Results:**

Sixty-three patients participated, median age 64.0 (interquartile range (IQR) 52.3–72.8) years, median hemoglobin concentration before transfusion 7.4 (IQR 7.1–7.7) g/dL. Median mitoPO_2_ values were 55.0 (IQR 49.6–63.0) mmHg before RBC transfusion, 51.0 (IQR 41.5–61.2) directly after and 67.3 (IQR 41.6–83.7) at 24 h after RBC transfusion. Median mitoVO_2_ values were 3.3 (IQR 2.1–5.9) mmHg/s before RBC transfusion, 3.7 (IQR 2.0–5.1) mmHg/s directly after, and 3.1 (IQR 2.5–4.8) mmHg/s 24 h after RBC transfusion. In the higher Hb concentration group (> 7 g/dL), we saw a dissociation of the effect of RBC transfusion on mitoPO_2_ versus on mitoVO_2_ values. MitoPO_2_ and mitoVO_2_ values were not associated with commonly used parameters of tissue perfusion and oxygenation.

**Conclusion:**

RBC transfusion did not alter mitoPO_2_ and mitoVO_2_ in critically ill patients with anemia. MitoPO_2_ and mitoVO_2_ values were not notably associated with Hb concentrations, parameters of severity of illness and markers of tissue perfusion or oxygenation. Given the high baseline value, it cannot be excluded nor confirmed whether RBC can improve low mitoPO_2_.

Trial registration number NCT03092297 (registered 27 March 2017)

**Supplementary Information:**

The online version contains supplementary material available at 10.1186/s40635-024-00646-3.

## Introduction

Anemia is highly prevalent in critically ill patients [1–3], with reported incidences during intensive care unit (ICU) stay as high as 66–98% [2–7]. Severe anemia can lead to diminished oxygen carrying capacity, cellular oxygen deficit and organ dysfunction contributing to organ failure, morbidity, and (cardiac) mortality [1–3]. Red blood cell (RBC) transfusions are given to solve a possible cellular oxygen deficit in critically ill patients, currently guided predominantly by hemoglobin (Hb) concentrations. However, this guidance may not be optimal since hemoglobin concentration is an indirect marker of cellular oxygenation. Commonly used surrogate markers of tissue perfusion and cellular oxygenation include mean arterial pressure (MAP), cardiac output (CO), central venous oxygen saturation (ScvO_2_), lactate concentration, and venous-to-arterial carbon dioxide difference (pCO_2_ gap). A limitation of these markers is that their correction is not directly correlated with improved cellular oxygenation or organ perfusion [8–10]. Markers of microcirculatory perfusion have shown that RBC transfusions result in recruitment of the microcirculation, particularly in those with low baseline values, but have not shown benefit in clinically significant outcomes [11–13]. A parameter that directly measures cellular oxygenation could provide more accurate guidance to RBC transfusions.

The Cellular Oxygen Metabolism (COMET) monitoring system, measures the mitochondrial oxygen tension (mitoPO_2_) non-invasively at the bedside [14]. The system uses the protoporphyrin IX-triplet state lifetime technique (PpIX-TSLT) [15, 16]. In a hemodilution study in pigs, oxygen deficit was observed in the skin of the anterior chest wall prior to other parameters of tissue perfusion [17]. This was corroborated in two case studies in humans after clonidine administration intraoperatively and during intraoperative blood loss [14, 18]. A parameter that is indirectly calculated from mitoPO_2_ values is mitochondrial oxygen consumption (mitoVO_2_) [14, 19, 20]. It has been suggested that mitoVO_2_ can give additional information regarding mitochondrial function and oxygen consumption [19]. We therefore postulated that the COMET device might be a valuable monitor for measurement of tissue oxygenation in critically ill patients with anemia.

The aim of this study was to describe mitoPO_2_ and mitoVO_2_ values as assessed with the COMET device in critically ill patients with anemia, before and after red cell transfusion, and to examine the association of these parameters with commonly used parameters of tissue perfusion and tissue oxygenation and with indicators of severity of critical illness, demographic and outcome characteristics.

## Methods

### Study design

A detailed overview of the design, procedure and protocol of this study was published elsewhere [21]. A concise overview regarding the study design, data collection, PpIX-TSLT technique description and study procedure is given in the supplementary material. In short, we performed an observational cohort study between March 2018 and April 2020 in two academic ICU departments in the Netherlands. Critically ill patients with anemia, defined as an Hb concentration < 10 g/dL, with an arterial catheter in situ receiving RBC transfusion were included in the study. Critically ill patients in need of RBC transfusion within 4 h were excluded from the study, as well as critically ill patients with an expected admittance in the ICU unit < 24 h. Patients younger than 18 years, with a brown plaster allergy, with photodermatosis and/or porphyria or insufficient Dutch language comprehensibility were not deemed eligible for the study, as well as pregnant women.

### MitoPO_2_ and mitoVO_2_ measurements

MitoPO_2_ measurements consisted of two phases: first we did dynamic measurements, after which we performed static measurements. During the dynamic phase, local pressure was applied on the measurement probe resulting in occlusion of the underlying microcirculation, resulting in an immediate drop in mitoPO_2_ values as well as a fast recovery after release of the pressure [14, 21]. The mitoPO_2_ value was measured every second for 120 s. The mitoPO_2_ values before and during local pressure were used to fit a sigmoid function, after which a linear function was used to calculate the mitoVO_2_ on the steepest part of the sigmoid curve. After the dynamic phase, mitoPO_2_ was measured once per minute for five minutes, to obtain a mean mitoPO_2_ at each time point. More details regarding the study procedure is given in the supplementary material.

### Statistical analyses

Descriptive statistics were used to describe the characteristics of the study population. Quantitative data were shown as means with a standard deviation (SD) or median with an interquartile range (IQR), as appropriate. Categorical variables were presented as number (percentage). The number of observations that were missing were visualized and described. Number of mitoPO_2_ measurement moments without a valid value are presented in Supplementary Materials-Table 2 [22].

The mitoPO_2_ measurements and signal quality were described per measurement moment in the total population, and in subgroups of participating study centers. MitoPO_2_ and mitoVO_2_ values were described for the total study population and stratified according to pre-transfusion hemoglobin concentration of ≤ 7 g/dL and > 7 g/dL. We calculated the change in mitoPO_2_ at various timepoints after transfusion compared with before transfusion, for all individuals, as well as per pre-transfusion subgroups, and presented mean differences with 95% confidence intervals using non-missing mitoPO_2_ values at each measurement time point.

The non-missing mitoPO_2_ values before RBC transfusion were used to assess the association between mitoPO_2_ and mitoVO_2_ with demographic and outcome characteristics. Univariate analyses were performed using ANOVA to calculate the associations between mitoPO_2_ and demographic and outcome characteristics. To examine the association between hemodynamic characteristics and mitoPO_2_, mitoPO_2_, values of all measurement moments were used. Univariate analyses with ANOVA were used to calculate the associations between mitoPO_2_ and mitoVO_2_ with markers of tissue perfusion and oxygenation. It has been suggested that normal mitoPO_2_ values are between 40 and 70 mmHg [23, 24]. Therefore, mitoPO_2_ values were categorized into three subgroups: < 40 mmHg, 40–70 mmHg, and > 70 mmHg. Within these subgroups, markers of tissue perfusion and oxygenation were described, including a lactate concentration measured one measurement time later. Furthermore, the mitoPO_2_ values before RBC transfusion were categorized into the same subgroups to describe the course over time in these subgroups.

We used mitoPO_2_ values with a signal quality of at least 20% for our analyses. The cut-off value of the signal quality of a mitoPO_2_ measurement to ascertain a valid measurement is around 20%. We performed sensitivity analyses post hoc to evaluate the effect of less strict signal quality of measurements in our results. During the sensitivity analyses, all aforementioned analyses were performed with mitoPO_2_ values based on a signal quality of at least 10%.

MitoVO_2_ calculations were performed using an automated MATLAB (Mathworks, R2022b Update 3) algorithm [25]. All other statistical analyses were performed using R (R foundation for Statistical Computing, Vienna, Austria) [26].

## Results

### Characteristics of the study population

**Fig. 1:**
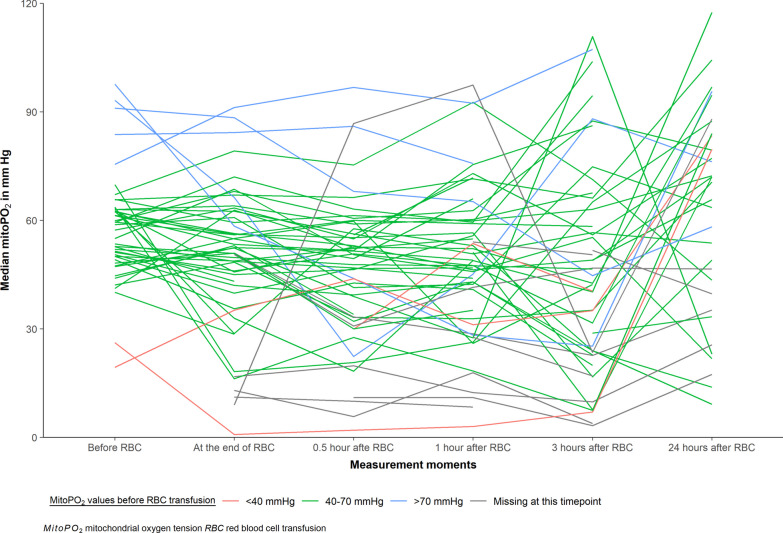
Spaghetti plot showing all observed valid (signal quality > 20%) mitoPO_2_ values measured with the COMET measurement device in all 63 critically ill patients with anemia, before and at various timepoints during the first 24 h after RBC transfusion. The range of mitoPO_2_ values is approximately 40–70 mmHg before RBC transfusion, while 24 h after RBC transfusion a wider range in mitoPO_2_ values is seen (15–110 mmHg). Red lines represent patients whose first valid mitoPO_2_ value was < 40 mmHg; green lines represent patients whose first valid mitoPO_2_ value was between 40 and 70 mmHg and blue line represents patients whose first valid mitoPO_2_ was above 70 mmHg

Of the 475 critically ill patients planned to receive RBC transfusion during the study period, 63 patients were included in the analyses, as depicted in Supplementary Materials-Fig. 1, corresponding to 378 observation moments (six measurements per included patient). Table 1 shows characteristics of the study population consisting of mostly male (76%), with a median age of 64.0 (IQR 53.0–73.0) years. The median Hb concentration before RBC transfusion was 7.4 (IQR 7.1; 7.7) g/dl. The median Hb concentration one hour after RBC transfusion was 8.2 (IQR 7.9; 8.9) g/dL, and it was 8.4 (IQR 7.9; 9.0) g/dL after 24 h.

**Table 1:** Characteristics of the study population of all 63 critically ill patients with anemia

Characteristic	Overall cohort (*n* = 63)
Age in years, median (IQR)	64.0 (53.0; 73.0)
Female sex, *n* (%)	15 (24%)
BMI in kg/m^2^, median (IQR)	27.9 (23.9; 32.6)
Admission reason, *n* (%)	
* Surgical*	31 (49.0%)
Cardiovascular	15 (48.4%)
Digestive	6 (19.4%)
Neurosurgery	2 (6.5%)
Genitourinary	1 (3.2%)
Trauma	3 (9.7%)
Miscellaneous	4 (12.9%)
Infectious process in throat	1 (3.2%)
Liver transplantation	1 (3.2%)
Arthrotomy	1 (3.2%)
Fasciitis necroticans	1 (3.2%)
* Non-surgical*	32 (51.0%)
Cardiovascular	2 (6.3%)
Respiratory	12 (37.5%)
Digestive	7 (21.9%)
Genitourinary tract	1 (3.1%)
Sepsis	8 (25.0%)
Neurological	1 (3.1%)
Chronic comorbidities, *n* (%)	
No comorbidity	22 (35.0%)
Chronic cardiovascular insufficiency	5 (8.0%)
Chronic obstructive lung disease	1 (2.0%)
Chronic renal insufficiency	2 (3.0%)
Chronic dialysis	1 (2.0%)
Cirrhosis	5 (8.0%)
Hematologic malignancy	3 (5.0%)
Immunologic deficiency	2 (3.0%)
Diabetes mellitus	22 (35.0%)
SOFA score before transfusion, median (IQR)	10 (8–12)
APACHE IV score, median (IQR)	70.0 (58.0; 88.0)
	*Missing: 2 (3.2%)*
Hemoglobin concentration before transfusion in g/dL, median (IQR)	7.4 (7.1; 7.7)
Hematocrit before transfusion in L/L, median (IQR)	0.23 (0.22; 0.24)
Days admitted to ICU at inclusion, median (IQR)	6.0 (3.0; 11.0)

*APACHE* Acute Physiologic and Chronic Health Evaluation, *BMI* body mass index, *IQR* interquartile range, *SOFA* Sequential Organ Failure Assessment

### MitoPO_2_ and mitoVO_2_ before and after RBC transfusion

Figure 1 presents all observed valid (signal quality > 20%) mitoPO_2_ values as assessed with the COMET measurement device in all 63 critically ill patients with anemia before and at the predefined timepoints during the first 24 h after RBC transfusion. MitoPO_2_ values prior to RBC transfusion showed large variation and were largely within normal limits. The overall median mitoPO_2_ before RBC transfusion was 55.0 (IQR 49.6; 63.0) mmHg, it was 51.0 (IQR 41.5; 61.2) mmHg at the end of the transfusion and 67.3 (IQR 41.6; 83.7) mmHg 24 h after RBC transfusion (Table 2). Stratification according to baseline mitoPO_2_ also yielded a heterogeneous response immediately following RBC transfusion (Fig. 1). After 24 h, those with a low baseline mitoPO_2_ < 40 mmHg tended to increase, whereas those with a mitoPO_2_ baseline value > 70 mmHg tended to decrease. However, the number of missing values at t = 24 h hamper statistical interpretation of this observation (supplementary materials-Table 3). Also, only two patients had mitoPO_2_ values below 30 mmHg.

**Table 2:** Mitochondrial oxygenation tension measured with COMET in critically ill patients receiving RBC transfusion in the total study population and stratified according to pre-transfusion hemoglobin concentration

Measurement time	MitoPO_2_^a^ in mmHg in all critically ill patients, median (IQR); *n*	MitoPO_2_^a^ in mmHg in strata of critically ill patients with anemia based on Hb concentration before RBC transfusion, median (IQR), n	
		Hb concentration ≤ 7 g/dL, *n* = 12	Hb concentration > 7 g/dL, *n* = 51
Before RBC transfusion	55.0 (49.6; 63.0); 51	61.3 (51.0; 69.3); 8	55.0 (48.9; 62.8); 42
End of RBC transfusion	51.0 (41.5; 61.2); 54	48.8 (23.1; 61.4); 11	51.0 (43.6; 60.4); 43
0.5 h after RBC transfusion	49.4 (33.2; 57.7); 55	51.6 (41.2; 79.3); 11	47.8 (32.6; 56.1); 44
1 h after RBC transfusion	47.5 (33.4; 59.4); 55	58.0 (44.5; 75.6); 11	47.1 (32.4; 55.3); 44
3 h after RBC transfusion	45.6 (23.8; 63.6); 50	59.6 (23.3; 88.3); 9	43.7 (24.0; 58.4); 41
24 h after RBC transfusion	67.3 (41.6; 83.7); 44	39.7 (25.6; 56.2); 6	71.3 (46.7; 83.9); 38

^a^MitoPO_2_ mitochondrial oxygen tension measured with the COMET system

Supplementary Materials-Table 2 presents the number of patient-moments at which we did not obtain a valid mitoPO_2_ value along with reasons for it. Absence of a valid mitoPO_2_ value was associated with severity of illness. The median APACHE IV score was 86.5 (IQR 59.3; 97.3) in critically ill patients without a valid mitoPO_2_, whereas the median APACHE IV score was 70.0 (IQR 58.0; 83.0) in the critically ill patients with a valid mitoPO_2_ value (Supplementary Materials-Table 4). In patients with a high SOFA score, mitoPO_2_ values tended to increase after RBC over time, whereas in those with a low SOFA score, mitoPO_2_ tended to decrease over time (Supplementary Table 6).

Twelve patients had a pre-transfusion Hb concentration ≤ 7 g/dL and their median mitoPO_2_ before RBC transfusion was 61.3 (IQR 51.0; 69.3) mmHg, while in patients with an Hb concentration > 7 g/dL it was 55.0 (IQR 48.9; 62.8) mmHg (Table 2). This is contrary to our hypothesis. The course of median mitoPO_2_ over time, stratified to pre-transfusion Hb concentration, suggests a decrease in those with low pre-transfusion Hb level (≤ 7 g/dL) and an increase in those with a higher pre-transfusion Hb level (> 7 g/dL) (Table 2 and Supplemental Materials-Fig. 3). Mean differences between mitoPO_2_ before RBC transfusion and mitoPO_2_ values at each measurement moment after RBC transfusion are depicted in Supplementary Materials-Table 5.

Supplementary Materials-Tables 8–16 present mitoPO_2_ values over time in a number of other subgroups, including stratification according to age or sex. The estimates were all consistent with our primary analysis.

If we look at O_2_ consumption, patients with a pre-transfusion Hb concentration of ≤ 7 g/dL had a median mitoVO_2_ before RBC transfusion of 7.6 (IQR 4.6; 10.5) mmHg/s. In patients with an Hb concentration > 7 g/dL pre-transfusion mitoVO_2_ was 3.3 (IQR 2.2; 5.7) mmHg/s (Supplementary Materials-Table 17).

Following RBC transfusion both mitoPO_2_ and mitoVO_2_ decreased in the low Hb concentration group (≤ 7 g/dL) whereas in the higher Hb concentration group (> 7 g/dL), we saw that after RBC transfusion, mitoPO_2_ increased while mitoVO_2_ did not change, i.e., a dissociation of the effect of RBC transfusion on mitoPO_2_ versus on mitoVO_2_ (Table 2, Supplementary Materials-Table 17).

### Association with clinical characteristics and with commonly used markers of tissue perfusion or oxygenation

Table 3 shows similar mitoPO_2_ and mitoVO_2_ values according to different demographic and clinical outcome characteristics. MitoPO_2_ per APACHE IV score: median mitoPO_2_ was 61.1 (IQR 59.0; 83.9) mmHg in the APACHE IV score < 50 subgroup; it was 53.1 (IQR 46.3; 62.5) mmHg in the APACHE IV score 50–80 subgroup, and 55.0 (50.5; 63.6) mmHg in the APACHE score > 80 subgroup (p-value for the trend = 0.137). Of all 378 mitoPO_2_ measurements 79 values were < 40 mmHg, and 169 mitoPO_2_ measurements were between 40 and 70 mmHg, and 53 mitoPO_2_ measurement > 70 mmHg (Table 4). Very low mitoPO_2_ (< 40 mmHg) was not convincingly associated with any of the measured markers of tissue perfusion and oxygenation (Table 4). Supplementary Materials-Table 19 similarly illustrates the absence of clear association between mitoPO_2_ or mitoVO_2_ values and the other measured markers of tissue perfusion and oxygenation. A statistically non-significant increase was seen in mitoVO_2_ values in patients with higher FiO_2_ values as depicted by a median mitoVO_2_ of 2.92 (IQR 2.01; 4.25) mmHg/s with FiO_2_ ≤ 30% to median 3.47 (IQR 2.30; 4.95) mmHg/s with FiO_2_ > 30% (*p*-value for trend = 0.074).

**Table 3:** Median mitoPO_2_ and mitoVO_2_ values, using the measurement before RBC transfusion, according to demographic and outcome characteristics of the study population

Characteristics	Number of participants with mitoPO_2_ measurement (total 51)^a^	MitoPO_2_ in mmHg, median (IQR)	Mean difference (95% CI)	*p*-value for trend	Number of participants with mitoVO_2_ measurement (total 26)^b^	MitoVO_2_ in mmHg/s, median (IQR)	Mean difference (95% CI)	*p*-value for trend
Demographic characteristics								
Sex								
Female	12	52.3 (33.5; 68.9)	Reference	0.742	4	1.99 (1.60; 3.00)	Reference	0.210
Male	39	50.9 (40.2; 63.0)	− 1.6 (− 11.3;8.1)		22	3.70 (2.34; 7.03)	2.56 (− 1.53; 6.65)	
Age								
< 50 years	7	56.9 (47.4; 62.4)	Reference	0.743	4	2.83 (1.92;4.94)	Reference	0.673
50–64 years	21	52.8 (41.6; 66.3)	− 1.5 (− 14.4; 11.5)		11	3.87 (2.23; 6.70)	0.97 (− 3.64; 5.58)	
65–75 years	15	47.0 (27.3; 58.1)	− 4.9 (− 18.6; 8.7)		8	3.98 (2.54; 7.13)	1.58 (-3.26; 6.42)	
> 75 years	8	51.0 (42.3; 62.5)	− 6.6 (− 22.0; 8.8)		3	2.88 (1.93; 3.40)	− 1.44 (− 7.47; 4.60)	
BMI								
< 25 kg/m^2^	19	56.1 (41.3; 66.3)	Reference	0.232	9	3.91 (2.12; 5.09)	Reference	0.547
25–30 kg/m^2^	14	49.4 (28.8; 60.1)	− 8.8 (− 19.0; 1.4)		7	3.09 (2.85; 4.74)	− 0.51 (− 4.43; 3.40)	
> 30 kg/m^2^	18	49.1 (40.4; 60.6)	− 3.1 (− 12.7; 6.3)		10	2.92 (1.81; 10.32)	1.41 (-2.16; 4.98)	
APACHE IV score								
< 50	6	61.1 (59.0; 83.9)	Reference	0.137	3	10.70 (7.81; 11.25)	Reference	0.102
50–80	26	53.1 (46.3; 62.5)	− 13.3 (− 26.5; − 0.1)		14	2.88 (2.21; 3.90)	− 4.91 (− 9.59; − 0.24)	
> 80	17	55.0 (50.5; 63.6)	− 10.0 (− 23.9; 3.8)		8	3.39 (1.59; 6.36)	− 4.92 (− 9.90; 0.06)	
Missing	*2*	*55.1 (52.7; 57.5)*			1	3.53 (3.53; 3.53)		
Reason of admission to ICU								
Medical	26	52.5 (43.9; 63.0)	Reference	0.536	10	2.24 (1.80; 4.67)	Reference	0.230
Surgical	25	49.8 (28.6; 63.5)	− 2.6 (− 10.8; 5.7)		16	3.70 (2.87; 7.20)	1.82 (− 1.23; 4.86)	
Hemoglobin concentration before RBC transfusion								
< 7 g/dL	7	52.1 (39.5; 75.7)	Reference	0.380	2	7.59 (4.64; 10.55)	Reference	0.269
≥ 7 g/dL	44	50.9 (39.3; 62.8)	− 5.2 (− 17.2; 6.7)		24	3.31 (2.16; 5.72)	− 3.06 (− 8.65; 2.52)	
Days admitted to ICU before inclusion								
< 6	22	50.5 (39.0; 63.1)	Reference	0.675	12	2.98 (1.55; 5.99)	Reference	0.670
≥ 6	29	52.3 (39.6; 63.4)	1.7 (− 6.6; 10.1)		14	3.89 (2.43; 5.92)	0.64 (− 2.42; 3.69)	
Outcome characteristics								
SOFA score before RBC transfusion								
< 10	17	47.0 (27.8; 56.5)	Reference	0.365	9	5.61 (1.69; 10.70)	Reference	0.289
≥ 10	34	54.9 (42.8; 65.9)	3.9 (− 4.7; 12.6)		17	3.09 (2.30; 4.93)	− 1.65 (− 4.78; 1.49)	
SOFA score change in 24 h								
≥ 0 (decrease in SOFA score)	16	50.5 (28.7; 61.9)	Reference	0.297	13	3.53 (2.18; 6.03)	Reference	0.842
< 0 (increase in SOFA score)	28	53.0 (42.0; 53.5)	5.0 (− 14.7; 4.6)		11	2.88 (1.90; 5.35)	0.30 (− 2.78; 3.37)	
Missing	*7*	50.5 (48.7; 52.8)			2	7.32 (5.07; 9.56)		
Total ICU admission duration								
< 5 days	7	56.2 (43.1; 66.2)	Reference	0.547	2	6.56 (3.94; 9.18)	Reference	0.824
5–10 days	8	46.8 (27.6; 59.4)	1.7 (− 13.6; 16.9)		6	4.35 (2.90; 8.29)	− 1.10 (− 7.65; 5.44)	
11–20 days	15	50.8 (43.0; 63.2)	7.4 (− 6.1; 20.9)		5	2.88 (2.30; 4.93)	− 1.86 (− 8.57; 4.85)	
> 20 days	21	52.5 (35.1; 63.4)	7.5 (− 5.4; 20.3)		13	3.53 (2.12; 5.09)	− 2.36 (− 8.45; 3.73)	
Total hospital admission duration								
< 10 days	6	59.4 (51.4; 67.3)	Reference	0.648	2	6.56 (3.94; 9.18)	Reference	0.926
10–20 days	10	45.8 (33.0; 52.8)	0.4 (− 14.8; 15.7)		6	4.35 (2.50; 8.29)	− 1.19 (− 7.91; 5.52)	
21–30 days	9	56.3 (44.2; 67.6)	7.3 (− 8.3; 22.8)		5	2.18 (1.69; 4.93)	− 2.26 (− 9.13; 4.62)	
31–50 days	10	59.2 (49.3; 63.8)	4.7 (− 10.6; 20.0)		4	2.85 (2.65; 4.00)	− 2.76 (− 9.89; 4.36)	
> 50 days	16	51.2 (41.5; 62.7)	7.9 (− 6.2;22.1)		9	3.87 (2.88; 5.09)	− 1.90 (− 8.33; 4.53)	
In-hospital or ICU mortality								
No	37	51.7 (40.1; 63.5)	Reference	0.878	16	3.31 (2.01; 5.33)	Reference	0.925
Yes	14	50.9 (35.0; 62.9)	0.7 (− 8.5; 10.0)		10	3.90 (2.21; 6.92)	0.14 (− 3.00; 3.29)	

*APACHE* Acute Physiology and Chronic Health Evaluation, *BMI* body mass index*, CI* confidence interval, *Hb* hemoglobin, *ICU* intensive care unit, *MitoPO*_*2*_ mitochondrial oxygen tension measured with the COMET system, *MitoVO*_*2*_ mitochondrial oxygen consumption calculated with a linear function on a fitted sigmoid curve of the mitoPO_2_ measurements

^a^Number of participants with mitoPO_2_ measurements before RBC transfusion

^b^Number of participants with mitoVO_2_ measurements before RBC transfusion

**Table 4:** Characteristics of clinically used surrogate markers of tissue perfusion and oxygenation in critically ill patients in ICU within subgroups of mitoPO_2_ values of < 40, 40–70, and > 70 mmHg

Clinical characteristics^a^	MitoPO_2_		
	< 40 mmHg (*n* = 79)	40–70 mmHg (*n* = 169)	> 70 mmHg (*n* = 53)
MitoPO_2_ in mmHg, median (IQR)	23.5 (13.5; 31.0)	52.8 (47.5; 59.9)	86.2 (75.7; 94.6)
MitoVO_2_ in mmHg/s, median (IQR) *Missing, n (%)*	2.44 (1.54; 3.75) *26 (32.9%)*	3.15 (2.20; 4.91) *86 (50.9%)*	4.13 (3.00; 5.36) *15 (28.3%)*
MAP in mmHg, median (IQR)	79.0 (69.2; 87.7)	80.0 (72.7; 89.0)	77.0 (71.0; 87.7)
Vasopressor use, *n* (%) *Missing, n (%)*	39 (49%) *0 (0.0%)*	93 (55%) *1 (0.6%)*	19 (36%) *0 (0.0%)*
Lactate in mmol/L, median (IQR) *Missing, n (%)*	1.5 (1.1; 1.9) *3 (3.8%)*	1.4 (1.0; 1.7) *12 (7.1%)*	1.5 (1.2; 1.9) *6 (11.3%)*
Lactate one measurement later in mmol/L, median (IQR) *Missing, n (%)*	1.5 (1.2; 1.9) *17 (21.5%)*	1.4 (1.1; 1.7) *35 (20.7%)*	1.4 (1.0; 1.8) *32 (60.4%)*
ScvO_2_ in %, median (IQR) *Missing, n (%)*	70.0 (64.5.0; 75.0) *32 (40.5%)*	71.0 (64.0; 76.8) *79 (46.7%)*	69.7 (59.3; 80.5) *23 (43.4%)*
pCO_2_ gap in mmHg, median (IQR) *Missing, n (%)*	4.5 (2.3; 6.0) *41 (51.9%)*	4.5 (2.4; 6.6) *95 (56.2%)*	4.5 (3.0; 6.0) *28 (52.8%)*
Cardiac index in L/min/m^2^, median (IQR) *Missing, n (%)*	3.8 (3.1; 4.3) *48 (60.8%)*	3.9 (3.0; 5.2) *108 (63.9%)*	3.6 (3.1; 4.2) *32 (60.4%)*
Fractional inspired oxygen in %, median (IQR) *Missing, n (%)*	35.0 (30.0; 40.0) *15 (19%)*	35.0 (28.0; 45.0) *20 (11.8%)*	35.0 (25.0; 40.0) *8 (15.1%)*
Fluid balance in L, median (IQR) *Missing, n (%)*	0.749 (− 0.295; 1.927) *7 (8.9%)*	0.742 (− 0.018; 1.423) *37 (21.9%)*	0.582 (− 0.541; 1.105) *14 (26.4%)*

There were 77 mitoPO_2_ measurements in 37 patients missing, which were not used in the subgroups

*MitoPO*_*2*_ mitochondrial oxygen tension measured with the COMET system; *MitoVO*_*2*_ mitochondrial oxygen consumption calculated with a linear function on a fitted sigmoid curve of the mitoPO_2_ measurements; *pCO*_*2*_* gap* venous-to-arterial carbon dioxide difference; *ScvO*_*2*_ central venous oxygen saturation; *SOFA* Sequential Organ Function Assessment

^a^These are median values over the overall study period

### Sensitivity analyses

The results of the sensitivity analyses with mitoPO_2_ value with a signal quality of at least 10% showed similar results and trends as our main analyses where the signal quality used was at least 20% (Supplementary Materials-Tables 20–25).

## Discussion

We performed a study in which we assessed mitoPO_2_ and mitoVO_2_ in critically ill patients with anemia before and after RBC transfusion. MitoPO_2_ values in critically ill patients with anemia were not substantially lower than values previously observed in other critically ill patients and did not significantly change during the first 24 h after RBC transfusion. MitoPO_2_ and mitoVO_2_ values were not notably associated with Hb concentrations, parameters of severity of illness, and markers of tissue perfusion or cellular oxygenation in our study population. In patients with a pre-transfusion concentration > 7 g/dL we saw a dissociation between mitoPO_2_ and mitoVO_2_ with respect to the effect of RBC transfusion. In patients with a pre-transfusion Hb concentration ≤ 7 g/dL, both mitoPO_2_ and mitoVO_2_ did not increase after RBC transfusion but rather both decreased over time (24 h).

### Main findings in relation to what is already known about the topic

Our study showed relatively normal mitoPO_2_ values, in critically ill patients with anemia, that did not increase after a RBC transfusion. An absence of effect of RBC transfusion on mitoPO_2_ is in line with a previous trial showing no benefit of guiding RBC transfusion according to a marker of tissue oxygenation, although some observational studies have suggested benefit of this approach [27, 36, 37]. Theoretically one would expect an increase in tissue oxygenation after RBC transfusion in patients who benefit from transfusion, especially in the critically ill patient with markers of low tissue perfusion and low Hb concentration before RBC transfusion [11, 27]. However, most of our study participants had a transfusion trigger above 7 g/dL, as well as normal mitoPO_2_, lactate, MAP, ScvO_2_, and pCO_2_ gap values before RBC transfusion. These ‘normal’ indices of tissue perfusion before RBC transfusion, suggest that a too liberal transfusion trigger was used, which may possibly explain the absence of an increase in mitoPO_2_ values after RBC transfusion. This lack of effect caused by a too liberal transfusion trigger is supported by the fact that low mitoPO_2_ values are expected with hematocrit values of 0.14 L/L or lower [23], which none of our study participants had. Just one of our study participants received more than one RBC transfusion units before mitoPO_2_ measurements, thereby limiting the interpretation of our results to only critically ill patients receiving RBC transfusion. Furthermore, it might be suggested that the critical illness has not (yet) led to low mitoPO_2_ values. An alternative explanation may be that RBC transfusion is ineffective at improving tissue oxygenation in this cohort of critically ill patients which are not actively resuscitated. Pre-transfusion baseline values have previously been shown important in predicting the response to transfusion on a microcirculatory level. A recent study suggested that a critically low mitoPO_2_ value of 30 mmHg or lower would be indicative of tissue hypoxia [23]. Of note, only 2 of the 63 critically ill patients with anemia pre-transfusion had a mitoPO_2_ < 30 mmHg. All the above considerations raise the question if RBC transfusions were needed in most of our critically ill patients with anemia [11, 12]. A final explanation of an absence of effect of a single RBC transfusion on mitoPO_2_ might be that the effect of RBC transfusion might be too small to increase mitoPO_2_ value [21]. Being a new monitoring technique, multiple studies have already been performed with the COMET measurement device. Studies with the COMET measurement device in healthy volunteers and critically ill patients have shown a normal mitoPO_2_ ranging between 40 and 70 mmHg [24, 31–34]. A recent study into the mitochondrial oxygen measurement with the COMET measurement device has determined that a normal range of mitoPO_2_ in physiological steady state is between 40 and 60 mmHg in the skin [23]. Indeed, our median mitoPO_2_ values correspond with normal mitoPO_2_ values.

The mitoVO_2_ in our population ranged from 2.8 to 3.7 mmHg/s corresponding with mitoVO_2_ values between 3.3 and 4.6 mmHg/s in critically ill patients that have been described in other studies [31, 32]. This is lower than the mitoVO_2_ values found in healthy volunteers ranging from 5.8 to 6.7 mmHg/s [14, 33], suggesting a decreased cellular respiration in critically ill patients with anemia. Since mitoVO_2_ is not directly measured by the COMET measurement device, the mitoVO_2_ needs to be calculated from the mitoPO_2_ values during application of pressure on the COMET probe. Different mechanisms have been described to calculate the mitoVO_2_, using the Michaelis–Menten kinetics [14], fitting a sigmoid curve [32], or using a linear function [31]. These different approaches could lead to different results, therefore mitoVO_2_ comparison should be done cautiously.

Interestingly, when looking in patients with lower (≤ 7 g/dL) versus a higher (> 7 g/dL) pre-transfusion Hb concentration, we observed different baseline values of mitoPO_2_ and mitoVO_2_ and different effects of RBC on mitoPO_2_ and mitoVO_2_, that were unexpected. Contrary to our expectations, the patients with a pre-transfusion Hb concentration < 7 g/dL had somewhat higher mitoPO_2_ values compared to patients with Hb ≥ 7 g/dL. The higher mitoPO_2_ value could have been the result of mitochondrial adaptation for an optimal mitochondrial energy metabolism [9, 28, 29]. It has been described that oxygen consumption in the mitochondria can be reduced in response to mitochondrial hypoxia, leading to excess oxygen in the mitochondria. Inflammatory mediators, e.g., nitric oxide, in sepsis and shock have been described causing this mitochondrial adaptation [9, 28, 30]. However, this is contradicted by our finding that the calculated mitochondrial oxygen consumption was higher in in critically ill patients with a pre-transfusion Hb concentration < 7 g/dL compared to patients with Hb ≥ 7 g/dL. It would be interesting to study the activity of mitochondrial adaptation mechanisms and their influence on the mitoPO_2_ in future studies.

In the patients with a higher pre-transfusion Hb concentration, a dissociation between the effect of RBC on mitoPO_2_ and mitoVO_2_ was observed, i.e., mitoPO_2_ increased and mitoVO_2_ did not change after RBC. Concomitantly, we observed a decrease both in mitoPO_2_ and mitoVO_2_ after RBC in patients with a low pre-transfusion Hb concentration. A possible explanation that has been offered before may be the nitric oxide-dependent vasodilatation effect of RBC transfusions due to plasma-free Hb [27]. Furthermore, this may suggest an inability of cells to use oxygen, previously referred to as cellular dysoxia, as shown before in sepsis patients [28].

A relatively large part of the mitoPO_2_ values had a low signal quality, which persisted until one hour after the end of RBC transfusion. This has been reported in other studies using the COMET measurement device [31, 32, 35]. Importantly, this is one of the first studies describing the characteristics of the patients with missing mitoPO_2_ values due to signal quality below a protocol-set threshold. It seems that overall, these patients were more critically ill compared to the critically ill patients with valid mitoPO_2_ measurements, which may have led to an overestimation of mitoPO_2_. The critical illness might have influenced the ALA-plaster absorption or PpIX formation, resulting in a sub-par signal quality after four hours ALA plaster induction. Our data suggest that more than 4 h ALA plaster induction may be needed to guarantee adequate upregulation of PpIX in critically ill patients for a qualitative mitoPO_2_ measurement with the COMET measurement device.

### Strength and limitations

Strengths of our study entailed the prospective nature of our study in multiple study sites, as well as the gathering of data at multiple timepoints. The data gathering was made as complete as possible to gain as much insight into the critically ill patients with anemia. Furthermore, the study design mimics clinical practice, making it more applicable to the daily practice.

Despite the high protocol adherence, missing data could not be prevented. Overall, most missing data were due to logistical issues, i.e., measurement in a weekend day or night time when no one of the study team was available. Therefore, missing not at random could not be ruled out, hence missing data could not be handled with imputation methods. We therefore interpreted our data cautiously, keeping in mind the large confidence intervals of mitoPO_2_ values, while looking into the mean and median mitoPO_2_ values.

### Clinical implications

This study is one of the first studies looking into bedside cellular oxygenation in patients receiving RBC transfusion and the effect of this RBC transfusion on the cellular oxygenation. It shows that in critically ill patients, overall mitoPO2 values are normal, and that when administered based on an Hb trigger, RBC transfusion does not result in an increase in mitoPO_2_ or mitoVO_2_. Findings are in line with other studies trying to determine the efficacy of RBC transfusion on the level of tissue oxygenation. Whether results are due to a too liberal RBC transfusion policy, or to an inability to utilize oxygen, or to a decrease in perfusion, or to another cause, cannot be dissected from our findings. In follow-up studies on the utility of mitoPO_2_ to guide interventions to improve tissue oxygenation, it should be noted that signal quality is impaired in the most severely ill patients.

## Conclusion

MitoPO_2_ and mitoVO_2_ in critically ill patients in the ICU with anemia were similar to previously observed in critically ill patients and did not significantly change during the first 24 h after RBC transfusion. MitoPO_2_ and mitoVO_2_ values were not notably associated with Hb concentrations, parameters of severity of illness, markers of tissue perfusion or cellular oxygenation in these moderately ill ICU patients with anemia. Given the high baseline value, it cannot be excluded nor confirmed whether RBC can improve low mitoPO_2_ values.

### Supplementary Information

Additional file 1.

## Data Availability

The datasets used and/or analyzed during the current study are available from the corresponding author on reasonable request.
