# Supplementary material for: Mitochondrial oxygen tension in critically ill patients receiving red blood cell transfusions: a multicenter observational cohort study
Source: Intensive Care Med Exp. 2024 Jul 8;12:61. doi: 10.1186/s40635-024-00646-3 (PMC11231106; doi:10.1186/s40635-024-00646-3)
Supplement: Supplementary file 1 — Additional file 1. [file 40635_2024_646_MOESM1_ESM.docx]

# Supplementary file

## Mitochondrial oxygen tension in critically ill patients receiving red blood cell transfusions: a multicenter observational cohort study

**Authors:**

M. Baysan, MD^1,2,3^

B. Hilderink, MD^4,5^

L van Manen, MD, PhD^4^

C. Caram-Deelder, PhD^2^

E.G. Mik, MD, PhD^6^

N.P. Juffermans, MD, PhD^4,5,7^

J.G. van der Bom, MD, PhD^2^

M.S. Arbous, MD, PhD^1,2^

1. Department of Intensive Care Medicine, Leiden University Medical Center, Leiden, the Netherlands

2. Department of Clinical Epidemiology, Leiden University Medical Center, Leiden, the Netherlands.

3. Jon J van Rood Center for Clinical Transfusion Research, Sanquin/LUMC, Leiden, the Netherlands

4. Department of Intensive Care Medicine, Amsterdam UMC, Location AMC, Amsterdam, The Netherlands.

5. Department of Intensive Care Medicine, OLVG Hospital, Amsterdam, the Netherlands

6. Department of Anesthesiology, Laboratory of Experimental Anesthesiology, Erasmus MC- University Medical Center Rotterdam, Rotterdam, the Netherlands

7. Laboratory of Translational Intensive Care, Erasmus MC, Rotterdam, the Netherlands

**Corresponding author:**

M. Sesmu Arbous

Email: [M.S.Arbous@lumc.nl](mailto:M.S.Arbous@lumc.nl)

**Date**:

14-06-2024

**Version**:

2.0

Table of contents

**Supplemental information regarding methods** 3

**Supplemental figure 1**: Flowchart7

**Supplemental figure 2:** Spaghetti plot of mitoVO_2_ values over time8

**Supplemental figure 3:** Median (IQR) of mitoPO_2_ over time stratified to pre-transfusion Hb concentration9

**Supplemental table 1:** STROBE checklist10

**Supplemental table 2:** Reason of missing mitoPO_2_ measurements per measurement moment 12

**Supplemental table 3:** MitoPO_2_ over time stratified to baseline mitoPO_2_ values13

**Supplemental table 4:** Demographic characteristics of patients with and without missing mitoPO_2_ measurements 14

**Supplemental table 5:** MitoPO_2_ change from baseline mitoPO_2_ measurement over time stratified to pre-transfusion Hb concentration 15

**Supplemental table 6:** MitoPO_2_ over time stratified to SOFA score 16

**Supplemental table 7:** MitoPO_2_ over time stratified to SOFA score difference in 24 hours 16

**Supplemental table 8:** MitoPO_2_ over time stratified to sex of patient 17

**Supplemental table 9:** MitoPO_2_ over time stratified to age of patient 17

**Supplemental table 10:** MitoPO_2_ over time stratified to BMI of patient 18

**Supplemental table 11:** MitoPO_2_ over time stratified to APACHE IV score of patient 18

**Supplemental table 12:** MitoPO_2_ over time stratified to reason of admission to the ICU 19

**Supplemental table 13:** MitoPO_2_ over time stratified to days admitted to the ICU before inclusion in the study 19

**Supplemental table 14:** MitoPO_2_ over time stratified to total ICU admission duration20

**Supplemental table 15:** MitoPO_2_ over time stratified to total hospital admission duration 20

**Supplemental table 16:** MitoPO_2_ over time stratified to ICU or in-hospital mortality 21

**Supplemental table 17:** MitoVO_2_ over time stratified to pre-transfusion Hb concentration 22

**Supplemental table 18:** Signal quality of mitoPO_2_ measurements over time23

**Supplemental table 19:** Association between mitoPO_2_ and mitoVO_2_ measurements with markers of tissue perfusion 24

**Supplemental table 20:** Reason of no valid mitoPO_2_ measurements due to signal quality of less than 10% per measurement moment 27

**Supplemental table 21:** Demographic characteristics of patients with and without valid mitoPO_2_ measurements (signal quality of at least 10%) 27

**Supplemental table 22:** Course of mitoPO_2_ with a signal quality of at least 10% over time and stratified to pre-transfusion Hb concentration28

**Supplemental table 23:** Association of mitoPO_2_ (with a signal quality of at least 10%) and mitoVO_2_ values with demographic and outcome characteristics29

**Supplemental table 24:** Median value of clinically used surrogate markers of tissue perfusion and oxygenation in critically ill patients in ICU within subgroups of mitoPO_2_ values of <40, 40-70, and >70 mmHg with a median signal quality of at least 10%31

**Supplemental table 25:** Association between mitoPO_2_ (with a signal quality of at least 10%) and mitoVO_2_ measurements with markers of tissue perfusion32

**Supplemental information regarding Methods**

## Study design

In short, this was a multicenter observational cohort study in two academic hospital ICU departments in the Netherlands: Leiden University Medical Center, and Amsterdam University Medical Center, location AMC. The participating ICUs were both treating mixed medical and surgical patients. Enrollment for this study started in March 2018 in Leiden University Medical Center and July 2018 in Amsterdam University Medical Center, and ended in April 2020 in both centers. Statistical analysis was performed from January 2023 to May 2023. The study was approved by the institutional ethics committee of Leiden The Hague Delft (reference P16.303) and was conducted according to the declaration of Helsinki and its later amendments. The study was registered in ClinicalTrials.gov (NCT03092297) prior to study enrollment. Informed consent was initially sought as soon as a patient with an expected admission duration of more than a day was admitted to the ICU. However, a few eligible patients could not be included in the study due to logistical problems related to a timely informed consent procedure with the patient and/or their legal representative. Therefore, the informed consent procedure was optimized from February 2019 onwards with a deferred consent procedure, in which consent was sought within 48 hours after inclusion of the patient into the study. This amendment of the study was also approved by the institutional ethics committee. The study population consisted of critically ill patients with anemia, defined as a Hb concentration <10 g/dL, with an arterial catheter in situ receiving one or two RBC units. Patients younger than 18 years, in need of an RBC transfusion <4 hours, without a legal representative, with a brown plaster allergy, with photodermatosis and/or porphyria were not eligible for the study. Furthermore, patients with expected admittance <24 hours, pregnant women or patients without sufficient Dutch language comprehensibility were not eligible. Due to the longitudinal nature of data, in combination with the goal to describe mitoPO_2_ over time in critically ill patients receiving RBC transfusion, we pragmatically concurred that approximately 65 subjects should be sufficient for the intended analyses. The Strengthening the Reporting of Observational Studies in Epidemiology (STROBE) statement was used in the writing of this manuscript (supplemental table 1) [1].

Data collection

Additional data were collected from the hospital’s electronic patient file system by the coordinating investigator namely: age, sex, comorbidities, body mass index, reason of ICU admission, Sequential Organ Failure Assessment (SOFA) score on the day of RBC transfusion before RBC transfusion and the day after RBC transfusion [2,3], Acute Physiology and Chronic Health Evaluation (APACHE) IV [4,5] score, length of ICU and hospital stay, and mortality (both ICU and in-hospital). Furthermore, data regarding cardiac index, ScvO_2_, pCO_2_ gap, lactate, fluid balance, fractional inspired oxygen, vasopressor use, and MAP were collected at each mitoPO_2_ measurement moment. Cardiac index was assessed by a minimal invasive method, the Vigileo/FloTrac system [6].

Description of the PpIX-TSLT technique

The COMET measurement system (Photonics Healthcare, Utrecht, the Netherlands, CE marked) was used to assess mitoPO_2_. Description of the PpIX-TSLT technique in the COMET monitor has been published elsewhere [7-10]. The COMET device can be safely used in humans [10,11]. Only mitoPO_2_ measurements with a signal quality of at least 20% were deemed valid, because of a high noise at lower signal quality [10].

Study procedure

During working hours, the treating physicians immediately informed the investigators whenever they decided to prescribe RBC transfusion to a critically ill patient with anaemia (Hb <10 g/dL). Transfusion guidelines differed between the two participating centers: Physicians in the Leiden University Medical Center used Hb concentration, American Society of Anesthesiologist Classification, age of patient, cardiac state, pulmonary state, and volemic state for their decision to transfuse [12,13], resulting in hemoglobin transfusion thresholds between 6.5-9.7 g/dL; in the Amsterdam University Medical Center patients RBC transfusions were prescribed when their hemoglobin was lower than 7 g/dL [14]. RBC transfusions were neither delayed nor administered for the purpose of the study, and only the treating physician had the final decision in the RBC transfusion. This resulted in a study population consisting of critically ill patients with anemia receiving RBC transfusion with a median Hb of 7.4 (IQR 7.1; 7.7) g/dL. After assessment of eligibility criteria by the investigator and (deferred) informed consent, the patient was included in the study. The anterior chest wall of the patient was cleaned with alcohol, and a 5-aminolevulinic acid (ALA) containing patch of 8mg (Alacare, Photonamic GmbH, Wedel, Germany) was placed on the sternum. The RBC transfusion was given four hours after administration of the patch to secure that enough PpIX was formed. Four hours after induction of PpIX, the ALA patch was removed for a short period and mitoPO_2_ was measured with a probe of the COMET monitor that was placed lightly on the part of the skin where PpIX was induced.

The mitoPO_2_ measurement consisted of two phases: first we performed dynamic measurements and second, static measurements. The dynamic measurements, consisting of a mitoPO_2_ reading per second during 120 seconds, started with local occlusion of the microcirculation by applying manual pressure with the COMET probe on the exposed skin. An immediate drop in mitoPO_2_ value was expected due to blockade of microcirculatory oxygen supply despite the ongoing mitochondrial oxygen consumption, and witnessing such drop served as additional confirmation, next to signal quality, of the validity of the mitoPO_2_ readings. After release of the pressure on the microcirculation, recovery of the mitoPO_2_ value was expected [10,15]. Using the mitoPO_2_ values before and during the local occlusion of the microcirculation, mitoVO_2_ was calculated [16,17]. A sigmoid function was fitted on the values after which a linear function was used to assess the mitoVO_2_ on the steepest part of the sigmoid curve. After the dynamic phase, mitoPO_2_ was measured once per minute for five minutes, to obtain a mean mitoPO_2_ for that moment in time. During all measurement we turned off the room lights to minimize influences of different light conditions on mitoPO_2_ measurements (background noise) and PpIX utilization (photobleaching). Directly following the mitoPO_2_ measurement, the ALA patch was re-placed on the skin to protect the skin against phototoxicity. After completion of the last measurement (last timepoint), the exposed skin was protected from sunlight for an additional 24 hours with a plaster. The study measurements were performed before RBC transfusion, at the end of RBC transfusion, 0.5-hour, 1 hour, 3 hours, and 24 hours after end of RBC transfusion. During each study measurement, an arterial blood sample, and if applicable, a central venous blood gas was taken. Besides the study intervention, care was delivered according to local standard and protocolized practice in the participating centers.

## References

1. von Elm E, Altman DG, Egger M, Pocock SJ, Gotzsche PC, Vandenbroucke JP, Initiative S (2007) The Strengthening the Reporting of Observational Studies in Epidemiology (STROBE) statement: guidelines for reporting observational studies. Lancet (London, England) 370 (9596):1453-1457. doi:10.1016/S0140-6736(07)61602-X

2. Vincent JL, de Mendonca A, Cantraine F, Moreno R, Takala J, Suter PM, Sprung CL, Colardyn F, Blecher S (1998) Use of the SOFA score to assess the incidence of organ dysfunction/failure in intensive care units: Results of a multicenter, prospective study. Critical care medicine 26 (11):1793-1800. doi:Doi 10.1097/00003246-199811000-00016

3. Vincent JL, Moreno R, Takala J, Willatts S, De Mendonca A, Bruining H, Reinhart CK, Suter PM, Thijs LG (1996) The SOFA (Sepsis-related Organ Failure Assessment) score to describe organ dysfunction/failure. On behalf of the Working Group on Sepsis-Related Problems of the European Society of Intensive Care Medicine. Intensive Care Med 22 (7):707-710

4. Zimmerman JE, Kramer AA, McNair DS, Malila FM (2006) Acute Physiology and Chronic Health Evaluation (APACHE) IV: hospital mortality assessment for today's critically ill patients. Crit Care Med 34 (5):1297-1310. doi:10.1097/01.Ccm.0000215112.84523.F0

5. Brinkman S, Bakhshi-Raiez F, Abu-Hanna A, de Jonge E, Bosman RJ, Peelen L, de Keizer NF (2011) External validation of Acute Physiology and Chronic Health Evaluation IV in Dutch intensive care units and comparison with Acute Physiology and Chronic Health Evaluation II and Simplified Acute Physiology Score II. J Crit Care 26 (1):105 e111-108. doi:10.1016/j.jcrc.2010.07.007

6. Thiele RH, Bartels K, Gan TJ (2015) Cardiac Output Monitoring: A Contemporary Assessment and Review. Critical care medicine 43 (1):177-185. doi:10.1097/Ccm.0000000000000608

7. Baysan M, Arbous MS, Mik EG, Juffermans NP, van der Bom JG (2020) Study protocol and pilot results of an observational cohort study evaluating effect of red blood cell transfusion on oxygenation and mitochondrial oxygen tension in critically ill patients with anaemia: the INsufficient Oxygenation in the Intensive Care Unit (INOX ICU-2) study. BMJ Open 10 (5):e036351. doi:10.1136/bmjopen-2019-036351

8. Mik EG (2013) Measuring Mitochondrial Oxygen Tension: From Basic Principles to Application in Humans. Anesth Analg 117:834-846. doi:10.1213/ANE.0b013e31828f29da

9. Mik EG, Balestra GM, Harms FA (2020) Monitoring mitochondrial PO2: the next step. Curr Opin Crit Care 26 (3):289-295. doi:10.1097/MCC.0000000000000719

10. Ubbink R, Bettink MAW, Janse R, Harms FA, Johannes T, Munker FM, Mik EG (2017) A monitor for Cellular Oxygen METabolism (COMET): monitoring tissue oxygenation at the mitochondrial level. J Clin Monit Comput 31 (6):1143-1150. doi:10.1007/s10877-016-9966-x

11. Harms F, Stolker RJ, Mik E (2016) Cutaneous Respirometry as Novel Technique to Monitor Mitochondrial Function: A Feasibility Study in Healthy Volunteers. PloS one 11 (7):e0159544. doi:10.1371/journal.pone.0159544

12. Sibinga CS, Das PC, Fratantoni JC (1998) Alternative Approaches to Human Blood Resources in Clinical Practice. vol 33. Kluwer Academic Publisher, Dordrecht

13. Centraal BegeleidingsOrgaan(CBO). Richtlijn Bloedtransfusie. Utrecht: CBO; 2011. http://nvb-trip-symposium.nl/wp-content/uploads/2017/08/Richtlijnbloedtransfusie2011.pdf. Accessed December 04, 2019 Hebert PC, Wells G, Blajchman MA, Marshall J, Martin C, Pagliarello G, Tweeddale M, Schweitzer I, Yetisir E (1999) A multicenter, randomized, controlled clinical trial of transfusion requirements in critical care. Transfusion Requirements in Critical Care Investigators, Canadian Critical Care Trials Group. N Engl J Med 340 (6):409-417. doi:10.1056/nejm199902113400601

15. Ubbink R, Wefers Bettink MA, van Weteringen W, Mik EG (2021) Mitochondrial oxygen monitoring with COMET: verification of calibration in man and comparison with vascular occlusion tests in healthy volunteers. J Clin Monit Comput 35 (6):1357-1366. doi:10.1007/s10877-020-00602-y

16. Streng L, de Wijs CJ, Raat NJH, Specht PAC, Sneiders D, van der Kaaij M, Endeman H, Mik EG, Harms FA (2022) In Vivo and Ex Vivo Mitochondrial Function in COVID-19 Patients on the Intensive Care Unit. Biomedicines 10 (7). doi:10.3390/biomedicines10071746

17. Neu C, Baumbach P, Plooij AK, Skitek K, Gotze J, von Loeffelholz C, Schmidt-Winter C, Coldewey SM (2020) Non-invasive Assessment of Mitochondrial Oxygen Metabolism in the Critically Ill Patient Using the Protoporphyrin IX-Triplet State Lifetime Technique-A Feasibility Study. Front Immunol 11:757. doi:10.3389/fimmu.2020.00757

**Supplemental figure 1:** Flowchart of inclusion of critically ill patients with anemia**.** Eventually 63 patients were included with 6 measurements each resulting in a total of 378 observations

Total number of patients receiving RBC transfusion (n=475)

Excluded (n=248)

- RBC transfusion outside working hours (n=185)
- Informed consent not in time (n=45)
- Participance in other study (n=14)
- No study team member available (n=3)
- No legal representative for informed consent (n=1)

Excluded from analysis (n=18)

- No consent after deferred consent (n=6)
- No RBC transfusion given (n=3)
- No signal or bad signal quality measurements (n=4)
- No measurements performed due to logistical reasons (n=4)
- Due to loss of intravenous access not meeting inclusion criteria after initial inclusion (n=1)

Assessed for eligibility (n=227)

Excluded (n=146)

- Declining study participation (n=32)
- Missed (n=15)
- Not matching eligibility criteria (n=99)
  - RBC transfusion <4 hours (n=52)
  - Admittance <24 hours (n=19)
  - Language barrier (n=12)
  - RBC trigger >10 g/dL (n=9)
  - Allergy (n=4)
  - Pregnant (n=1)
  - Age <18 years (n=1)
  - No RBC transfusion given (n=1)

Analysed (n=63 participants with in total 378 observations)

- mitoPO_2_ <40 mmHg : n=33 (79 observations)
- mitoPO_2_ 40-70 mmHg: n=54 (169 observations)
- mitoPO_2_ >70 mmHg : n=30 (53 observations)
- missing mitoPO_2_ measurements : n=37 (77 observations)

In the study (n=81)

**Supplemental figure 2:** Spaghettiplot showing the course of mitoVO_2_ values measured with a linear function based on fitted sigmoid curve of the mitoPO_2_ measurements with the COMET measurement device in all 63 critically ill patients with anemia, before and at various timepoints durig the first 24 hours after RBC transfusion. The range of mitoVO_2_ values seem larger before RBC transfusion, while the range of mitoVO_2_ values seem to be more concentrated 24 hours after RBC transfusion. More importantly, there seems to be missing mitoVO_2_ values during all measurement timepoints

**
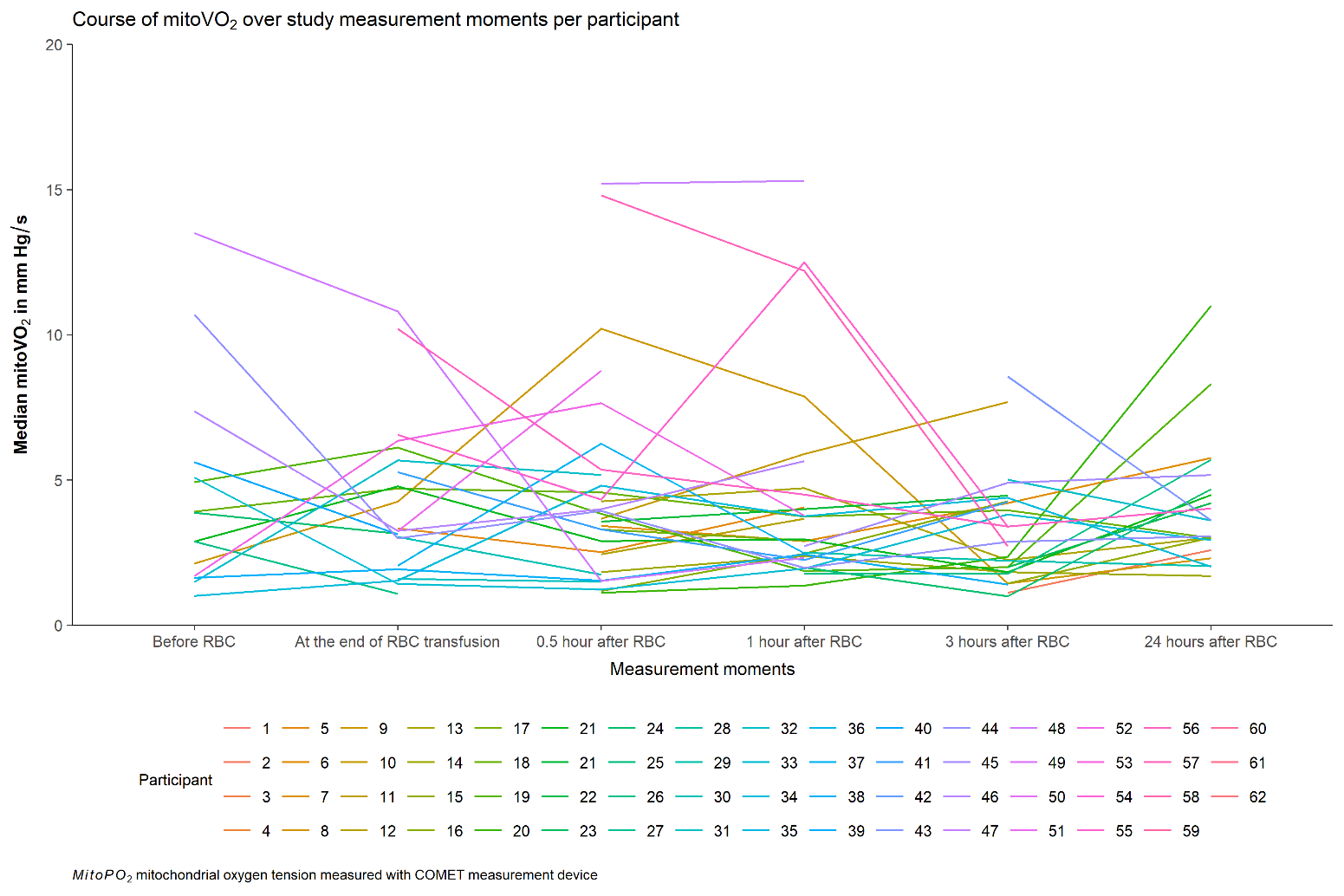
**

**Supplemental figure 3:** Median (IQR) of mitochondrial oxygen tension measured with COMET in critically ill patients receiving RBC transfusion stratified according to pre-transfusion hemoglobin concentration

**
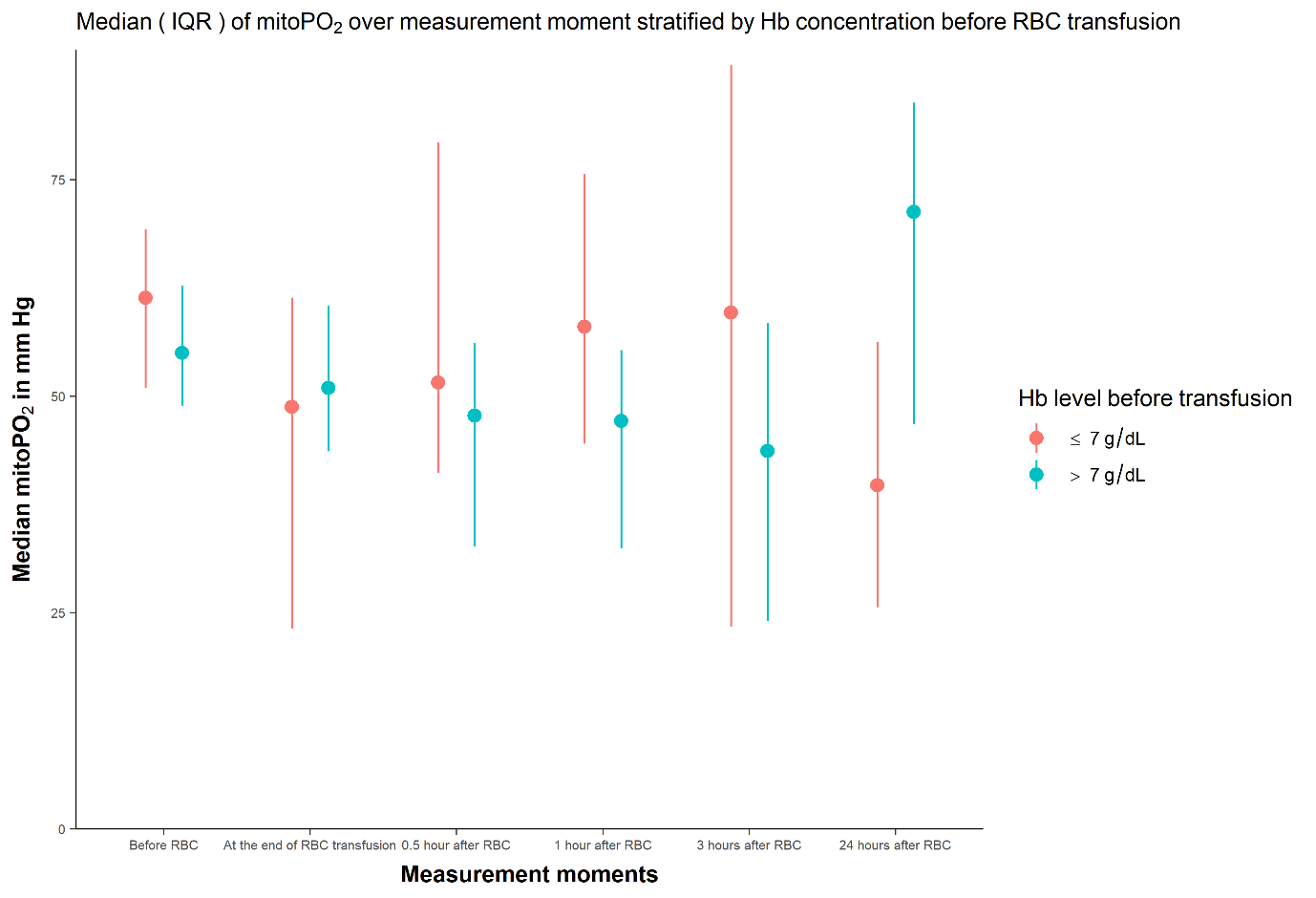
**

**Supplemental table 1:** STROBE checklist

# Reporting checklist for cohort study based on the STROBE cohort guidelines

|  |  | Reporting Item | | | Page Number |
| --- | --- | --- | --- | --- | --- |
| **Title and abstract** | | | | | |
| Title | | [#1a](https://www.goodreports.org/reporting-checklists/strobe-cohort/info/#1a) | Indicate the study’s design with a commonly used term in the title or the abstract | 1 | |
| Abstract | | [#1b](https://www.goodreports.org/reporting-checklists/strobe-cohort/info/#1b) | Provide in the abstract an informative and balanced summary of what was done and what was found | 2 | |
| **Introduction** | |  |  |  | |
| Background / rationale | | [#2](https://www.goodreports.org/reporting-checklists/strobe-cohort/info/#2) | Explain the scientific background and rationale for the investigation being reported | 3 | |
| Objectives | | [#3](https://www.goodreports.org/reporting-checklists/strobe-cohort/info/#3) | State specific objectives, including any prespecified hypotheses | 3 | |
| **Methods** | |  |  |  | |
| Study design | | [#4](https://www.goodreports.org/reporting-checklists/strobe-cohort/info/#4) | Present key elements of study design early in the paper | 4+ supplemental information | |
| Setting | | [#5](https://www.goodreports.org/reporting-checklists/strobe-cohort/info/#5) | Describe the setting, locations, and relevant dates, including periods of recruitment, exposure, follow-up, and data collection | Supplemental information | |
| Eligibility criteria | | [#6a](https://www.goodreports.org/reporting-checklists/strobe-cohort/info/#6a) | Give the eligibility criteria, and the sources and methods of selection of participants. Describe methods of follow-up. | Supplemental information | |
| Eligibility criteria | | [#6b](https://www.goodreports.org/reporting-checklists/strobe-cohort/info/#6b) | For matched studies, give matching criteria and number of exposed and unexposed | Supplemental information | |
| Variables | | [#7](https://www.goodreports.org/reporting-checklists/strobe-cohort/info/#7) | Clearly define all outcomes, exposures, predictors, potential confounders, and effect modifiers. Give diagnostic criteria, if applicable | Supplemental information | |
| Data sources / measurement | | [#8](https://www.goodreports.org/reporting-checklists/strobe-cohort/info/#8) | For each variable of interest give sources of data and details of methods of assessment (measurement). Describe comparability of assessment methods if there is more than one group. Give information separately for exposed and unexposed groups if applicable. | Supplemental information | |
| Bias | | [#9](https://www.goodreports.org/reporting-checklists/strobe-cohort/info/#9) | Describe any efforts to address potential sources of bias | n/a | |
| Study size | | [#10](https://www.goodreports.org/reporting-checklists/strobe-cohort/info/#10) | Explain how the study size was arrived at | n/a | |
| Quantitative variables | | [#11](https://www.goodreports.org/reporting-checklists/strobe-cohort/info/#11) | Explain how quantitative variables were handled in the analyses. If applicable, describe which groupings were chosen, and why | Supplemental information | |
| Statistical methods | | [#12a](https://www.goodreports.org/reporting-checklists/strobe-cohort/info/#12a) | Describe all statistical methods, including those used to control for confounding | 4+5 | |
| Statistical methods | | [#12b](https://www.goodreports.org/reporting-checklists/strobe-cohort/info/#12b) | Describe any methods used to examine subgroups and interactions | 4+5 | |
| Statistical methods | | [#12c](https://www.goodreports.org/reporting-checklists/strobe-cohort/info/#12c) | Explain how missing data were addressed | 4+5 | |
| Statistical methods | | [#12d](https://www.goodreports.org/reporting-checklists/strobe-cohort/info/#12d) | If applicable, explain how loss to follow-up was addressed | n/a | |
| Statistical methods | | [#12e](https://www.goodreports.org/reporting-checklists/strobe-cohort/info/#12e) | Describe any sensitivity analyses | n/a | |
|  | |  |  |  | |
| **Results** | |  |  |  | |
| Participants | | [#13a](https://www.goodreports.org/reporting-checklists/strobe-cohort/info/#13a) | Report numbers of individuals at each stage of study—eg numbers potentially eligible, examined for eligibility, confirmed eligible, included in the study, completing follow-up, and analysed. Give information separately for for exposed and unexposed groups if applicable. | 6 + supplemental fig 1 | |
| Participants | | [#13b](https://www.goodreports.org/reporting-checklists/strobe-cohort/info/#13b) | Give reasons for non-participation at each stage | Supplemental fig 1 | |
| Participants | | [#13c](https://www.goodreports.org/reporting-checklists/strobe-cohort/info/#13c) | Consider use of a flow diagram | Supplemental fig 1 | |
| Descriptive data | | [#14a](https://www.goodreports.org/reporting-checklists/strobe-cohort/info/#14a) | Give characteristics of study participants (eg demographic, clinical, social) and information on exposures and potential confounders. Give information separately for exposed and unexposed groups if applicable. | 6-7 + table 1 | |
| Descriptive data | | [#14b](https://www.goodreports.org/reporting-checklists/strobe-cohort/info/#14b) | Indicate number of participants with missing data for each variable of interest | 6-7 + supplemental tables 2-3 | |
| Descriptive data | | [#14c](https://www.goodreports.org/reporting-checklists/strobe-cohort/info/#14c) | Summarise follow-up time (eg, average and total amount) | n.a. | |
| Outcome data | | [#15](https://www.goodreports.org/reporting-checklists/strobe-cohort/info/#15) | Report numbers of outcome events or summary measures over time. Give information separately for exposed and unexposed groups if applicable. | 6-7 + tables 2-4 + supplemental tables 5-18 | |
| Main results | | [#16a](https://www.goodreports.org/reporting-checklists/strobe-cohort/info/#16a) | Give unadjusted estimates and, if applicable, confounder-adjusted estimates and their precision (eg, 95% confidence interval). Make clear which confounders were adjusted for and why they were included | 6-7+ tables 2-4 + supplemental table 18 | |
| Main results | | [#16b](https://www.goodreports.org/reporting-checklists/strobe-cohort/info/#16b) | Report category boundaries when continuous variables were categorized | 6-7 + tables 2-4 + supplemental figure 1+ supplemental tables 4-18 | |
| Main results | | [#16c](https://www.goodreports.org/reporting-checklists/strobe-cohort/info/#16c) | If relevant, consider translating estimates of relative risk into absolute risk for a meaningful time period | n/a | |
| Other analyses | | [#17](https://www.goodreports.org/reporting-checklists/strobe-cohort/info/#17) | Report other analyses done—eg analyses of subgroups and interactions, and sensitivity analyses | 6-7 + tables 2-4 + supplemental tables 19-23 | |
| **Discussion** | |  |  |  | |
| Key results | | [#18](https://www.goodreports.org/reporting-checklists/strobe-cohort/info/#18) | Summarise key results with reference to study objectives | 8-9 | |
| Limitations | | [#19](https://www.goodreports.org/reporting-checklists/strobe-cohort/info/#19) | Discuss limitations of the study, taking into account sources of potential bias or imprecision. Discuss both direction and magnitude of any potential bias. | 10 | |
| Interpretation | | [#20](https://www.goodreports.org/reporting-checklists/strobe-cohort/info/#20) | Give a cautious overall interpretation considering objectives, limitations, multiplicity of analyses, results from similar studies, and other relevant evidence. | 8-11 | |
| Generalisability | | [#21](https://www.goodreports.org/reporting-checklists/strobe-cohort/info/#21) | Discuss the generalisability (external validity) of the study results | 10 | |
| **Other Information** | |  |  |  | |
| Funding | | [#22](https://www.goodreports.org/reporting-checklists/strobe-cohort/info/#22) | Give the source of funding and the role of the funders for the present study and, if applicable, for the original study on which the present article is based | 12 | |

Notes:

- 14a: 6-7 + table 1 + supplemental table 4
- 14b: 6-7 + supplemental tables 2-3
- 15: 6-7 + tables 2-4 + supplemental tables 5-18
- 16a: 6-7+ tables 2-4 + supplemental table 18
- 16b: 6-7 + tables 2-4 + supplemental figure 1+ supplemental tables 4-18
- 17: 6-7 + tables 2-4 + supplemental tables 19-23

The STROBE checklist is distributed under the terms of the Creative Commons Attribution License CC-BY. This checklist was completed on 19. July 2023 using <https://www.goodreports.org/>, a tool made by the [EQUATOR Network](https://www.equator-network.org) in collaboration with [Penelope.ai](https://www.penelope.ai)

**Supplemental table 2:** Description of missing mitoPO_2_ measurement, per measurement moment and overall. Most missing at the start of the measurements was due to poor signal quality. Over time, missing is more due to logistical problems

| Measurement time | Missing mitoPO_2_ measurements,  n (% total)^1,2^ | Reason Missing |
| --- | --- | --- |
| Before RBC transfusion | 12 (19.0%) | - Signal quality <20% (n=12) |
| End of RBC transfusion | 11 (17.4%) | - Signal quality <20% (n=8) - Logistical problems (n=2) - Measurement data not saved (n=1) |
| 0.5hour after RBC transfusion | 10 (15.9%) | - Signal quality <20% (n=7) - Measurement data not saved (n=1) - Logistical problems (n=1) - Technical problems measurement device (n=1) |
| 1 hour after RBC transfusion | 9 (14.3%) | - Signal quality <20% (n=5) - Logistical problems (n=2) - Measurement data not saved (n=1) - Technical problems measurement device (n=1) |
| 3 hours after RBC transfusion | 15 (23.8%) | - Logistical problems (n=11) - Measurement data not saved (n=1) - Signal quality <20% (n=2) - Technical problems measurement device (n=1) |
| 24 hours after RBC transfusion | 20 (31.7%) | - Logistical problems (n=16) - Measurement data not saved (n=2) - Signal quality <20% (n=1) - Technical problems measurement device (n=1) |
| *Overall* | *77 (20.4%)* | - *Signal quality <20% (n=35)* - *Logistical problems (n=32)* - *Measurement data not saved (n=6)* - *Technical problems measurement device(n=4)* |

1 MitoPO_2_ mitochondrial oxygen tension measured with the COMET system

2 There were a total of 63 mitoPO_2_ measurements per measurement time, with in total 378 measurements

**Supplemental table 3:** Mitochondrial oxygenation tension (mitoPO_2_) measured with COMET in critically ill patients receiving RBC transfusion in the total study population and stratified according to the mitoPO_2_ values of the patient before RBC transfusion

| Measurement time | MitoPO_2_^1^ in mmHg in all critically ill patients,  median (IQR), n | MitoPO_2_^1^ in mmHg in strata of the mitoPO_2_ value before RBC transfusion,  median (IQR), n | | |
| --- | --- | --- | --- | --- |
|  |  | *<40 mmHg,*  *n=2* | *40-70 mmHg,*  *n=39* | *>70 mmHg,*  *n=5* |
| Before RBC transfusion | 55.0 (49.6; 63.0),  51 | 26.2 (22.8; 32.9),  2 | 55.0 (50.0; 62.5),  39 | 91.0 (83.8; 93.2),  5 |
| End of RBC transfusion | 51.0 (41.5; 61.2),  54 | 18.0 (9.4; 26.5),  2 | 52.4 (45.2; 60.4),  39 | 84.3 (66.3; 88.4),  5 |
| 0.5hr after RBC transfusion | 49.4 (33.2; 57.7),  55 | 29.9 (15.9; 36.9),  2 | 51.6 (41.7; 57.3),  39 | 68.0 (43.9; 86.0),  5 |
| 1 hr after RBC transfusion | 47.5 (33.4; 59.4),  55 | 31.1 (17.7; 42.4),  2 | 49.9 (43.0; 59.6),  36 | 65.20 (45.1; 75.7),  5 |
| 3 hr after RBC transfusion | 45.6 (23.8; 63.6),  50 | 35.0 (21.0; 37.7),  2 | 52.1 (30.9; 66.5),  33 | 66.4 (39.8; 92.9),  5 |
| 24 hr after RBC transfusion | 67.3 (41.6; 83.7),  44 | 81.6 (80.6; 82.5),  2 | 71.3 (47.7; 86.6),  30 | 76.2 (6792; 85.3),  4 |

1 MitoPO_2_ mitochondrial oxygen tension measured with the COMET system

**Supplemental table 4**: Overview of demographic characteristics of our study cohort with no missing mitoPO_2_ measurements and the study cohort with missing mitoPO_2_ measurements due to poor signal quality before RBC transfusion. The median BMI, APACHE IV score, and proportion of mortality were higher in the study cohort with missing mitoPO_2_ measurements before RBC transfusions. Interestingly, the median hemoglobin concentration is lower in this cohort compared to the study cohort with no missing mitoPO_2_ measurement before RBC transfusion

| Characteristic | Cohort with no missing mitoPO_2_ before RBC transfusion (n=51) | Cohort with missing mitoPO_2_ before RBC transfusion due to signal quality <20% (n=12) |
| --- | --- | --- |
| Age in years, median (IQR) | 64.0 (55.5-73.0) | 64.0 (52.0-70.8) |
| Female sex, n (%) | 12 (24%) | 3 (25%) |
| BMI, median (IQR) | 27.6 (24.0-32.4) | 27.8 (23.9-30.4) |
| Admission reason, n(%)  Surgical  Non-surgical | 25 (49%)  26 (51%) | 6 (50%)  6 (50%) |
| Chronic comorbidities, n(%)  No known comorbidity  Chronic Cardiovascular Insufficiency  Chronic Obstructive Lung Disease  Chronic Renal Insufficiency  Chronic dialysis  Cirrhosis  Hematologic malignancy  Immunologic deficiency  Diabetes mellitus | 19 (37%)  5 (10%)  1 (2%)  2 (4%)  0  1 (2%)  3 (6%)  2 (4%)  18 (35%) | 3 (25%)  0 (0%)  0 (0%)  0 (0%)  0 (0%)  4 (33%)  0 (0%)  0 (0%)  4 (33%) |
| SOFA score before transfusion, median (IQR) | 10.0 (8.5-11.0) | 10.5 (7.8-13.5) |
| APACHE IV score, median (IQR) | 70.0 (58.0-83.0) | 86.5 (59.3-97.3) |
| Hemoglobin concentration before transfusion in g/dL, median (IQR) | 7.6 (7.3-7.7) | 7.3 (6.9-7.7) |
| Hematocrit before transfusion in L/L, median (IQR) | 0.23 (0.22-0.24) | 0.22 (0.21-0.23) |
| Days admitted to ICU at inclusion, median (IQR) | 6.0 (3.0-11.5) | 3.5 (2.0-7.5) |
| Total ICU admission duration in days, median (IQR) | 16.0 (9.5-27.0) | 17.0 (8.0-35.5) |
| Total hospital admission duration in days, median (IQR) | 32.0 (18.0-62.0) | 34.5 (28.5-53.8) |
| ICU or in-hospital mortality, n(%) | 14 (27%) | 5 (42%) |

*APACHE* Acute Physiology and Chronic Health Evaluation; *BMI* Body mass index*; CI* confidence interval; *ICU* Intensive care unit; *SOFA* Sequential Organ Failure Assessment

**Supplemental table 5**: The change in mitoPO_2_ values after RBC transfusion compared to before RBC transfusion mitoPO_2_ value in the study population, as well as in subgroups of Hb concentrations before RBC transfusion. A small decrease in mitoPO_2_ values can be seen in both subgroups of Hb concentrations. Twenty-four hours after RBC transfusion, mitoPO_2_ measurements were missing in the subgroup of Hb concentration ≤7 g/dL

| Measurement time | Change in mitoPO_2_^1^ from mitoPO_2_ value before RBC transfusion in mmHg in the total cohort and in subgroups according to Hb concentration before RBC transfusion, median (IQR); n | | |
| --- | --- | --- | --- |
|  | *Overall,*  *n=63* | *Hb concentration ≤ 7 g/dL,*  *n=12* | *Hb concentration >7 g/dL,*  *n=51* |
| Before RBC transfusion | *reference* | *reference* | *reference* |
| End of RBC transfusion | -2.6 (-9.5; 6.1);  47 | 0.5 (-7.2; 4.4);  8 | -3.1 (-11.3; 5.9);  39 |
| 0.5 hour after RBC transfusion | -6.3 (-10.8; 0.6);  48 | -4.0 (-7.4; 4.5);  8 | -6.6 (-15.2; 0.1);  40 |
| 1 hour after RBC transfusion | -6.7 (-13.2; 3.7);  46 | -4.3 (-5.1; 9.2);  8 | -9.2 (-16.0; 0.1);  38 |
| 3 hours after RBC transfusion | -5.1 (-19.8; 4.4);  41 | 24.8 (4.6; 31.8);  6 | -5.3 (-20.0; 0.7);  35 |
| 24 hours after RBC transfusion | 17.6 (-12.5; 31.4);  36 | *missing* | 17.6 (-12.5; 31.4);  36 |

*Hb* Hemoglobin; *MitoPO_2_* mitochondrial oxygen tension measured with the COMET system; *RBC* Red blood cell

1 MitoPO_2_ mitochondrial oxygen tension measured with the COMET system

**Supplemental table 6:** Mitochondrial oxygenation tension measured with COMET in critically ill patients receiving RBC transfusion in the total study population and stratified according to the SOFA score of the patient before RBC transfusion

| Measurement time | MitoPO_2_^1^ in mmHg in all critically ill patients,  median (IQR), n | MitoPO_2_^1^ in mmHg in strata of critically ill patients with anemia according to SOFA score < or > 10 before RBC transfusion,  median (IQR), n | |
| --- | --- | --- | --- |
|  |  | *<10,*  *n=22* | *≥10.*  *n=41* |
| Before RBC transfusion | 55.0 (49.6; 63.0),  51 | 50.0 (46.0; 62.7),  17 | 59.1 (50.6; 63.5),  34 |
| End of RBC transfusion | 51.0 (41.5; 61.2),  54 | 49.8 (28.7; 52.5),  18 | 53.4 (44.1; 61.8),  36 |
| 0.5hr after RBC transfusion | 49.4 (33.2; 57.7),  55 | 46.7 (30.0; 56.3),  18 | 50.6 (38.1; 59.2),  37 |
| 1 hr after RBC transfusion | 47.5 (33.4; 59.4),  55 | 46.8 (27.5; 52.2),  18 | 48.9 (41.5; 59.9),  37 |
| 3 hr after RBC transfusion | 45.6 (23.8; 63.6),  50 | 35.1 (16.9; 49.1),  16 | 49.0 (25.2; 66.1),  34 |
| 24 hr after RBC transfusion | 67.3 (41.6; 83.7),  44 | 39.7 (24.3; 64.4),  16 | 74.2 (53.7; 89.7),  28 |

1 MitoPO_2_ mitochondrial oxygen tension measured with the COMET system

**Supplemental table 7:** Mitochondrial oxygenation tension measured with COMET in critically ill patients receiving RBC transfusion in the total study population and stratified according to the SOFA score difference over 24 hours of the patient

| Measurement time | MitoPO_2_^1^ in mmHg in all critically ill patients,  median (IQR), n | MitoPO_2_^1^ in mmHg in strata of critically ill patients with anemia according to SOFA score difference over 24 hours,  median (IQR), n | |
| --- | --- | --- | --- |
|  |  | *Decrease in SOFA score,*  *n=35* | *Increase in SOFA score.*  *n=20* |
| Before RBC transfusion | 55.0 (49.6; 63.0),  51 | 59.7 (50.2; 64.3),  28 | 56.1 (45.7; 61.6),  17 |
| End of RBC transfusion | 51.0 (41.5; 61.2),  54 | 52.7 (45.0; 61.2),  29 | 51.0 (29.8; 61.2),  19 |
| 0.5hr after RBC transfusion | 49.4 (33.2; 57.7),  55 | 49.4 (36.5; 60.3),  32 | 50.5 (20.7; 54.9),  18 |
| 1 hr after RBC transfusion | 47.5 (33.4; 59.4),  55 | 50.8 (41.9; 61.3),  31 | 48.9 (28.6; 56.5),  18 |
| 3 hr after RBC transfusion | 45.6 (23.8; 63.6),  50 | 49.8 (30.0; 67.4),  29 | 35.1 (19.7; 55.6),  16 |
| 24 hr after RBC transfusion | 67.3 (41.6; 83.7),  44 | 67.3 (46.9; 86.0),  28 | 63.4 (31.7; 77.8),  16 |

1 MitoPO_2_ mitochondrial oxygen tension measured with the COMET system

**Supplemental table 8:** Mitochondrial oxygenation tension measured with COMET in critically ill patients receiving RBC transfusion in the total study population and stratified according to the sex of the patient

| Measurement time | MitoPO_2_^1^ in mmHg in all critically ill patients,  median (IQR), n | MitoPO_2_^1^ in mmHg in strata of critically ill patients with anemia according to the sex of patient,  median (IQR), n | |
| --- | --- | --- | --- |
|  |  | *Female,*  *n=15* | *Male.*  *n=48* |
| Before RBC transfusion | 55.0 (49.6; 63.0),  51 | 56.2 (48.0; 64.3),  13 | 55.0 (50.0; 63.0),  37 |
| End of RBC transfusion | 51.0 (41.5; 61.2),  54 | 51.7 (36.9; 55.3),  15 | 50.8 (42.3;62.1),  39 |
| 0.5 hr after RBC transfusion | 49.4 (33.2; 57.7),  55 | 45.3 (30.5; 60.4),  15 | 49.4 (36.3; 56.1),  40 |
| 1 hr after RBC transfusion | 47.5 (33.4; 59.4),  55 | 49.0 (35.2; 62.7),  14 | 47.4 (32.8; 56.5),  41 |
| 3 hr after RBC transfusion | 45.6 (23.8; 63.6),  50 | 55.0 (28.1; 81.9),  11 | 43.7 (23.1; 58.1),  39 |
| 24 hr after RBC transfusion | 67.3 (41.6; 83.7),  44 | 61.3 (26.7; 87.6),  13 | 70.6 (46.9; 82.0),  31 |

1 MitoPO_2_ mitochondrial oxygen tension measured with the COMET system

**Supplemental table 9:** Mitochondrial oxygenation tension measured with COMET in critically ill patients receiving RBC transfusion in the total study population and stratified according to the age of the patient

| Measurement time | MitoPO_2_^1^ in mmHg in all critically ill patients,  median (IQR),n | MitoPO_2_^1^ in mmHg in strata of critically ill patients with anemia according to the age of patient,  median (IQR), n | | | |
| --- | --- | --- | --- | --- | --- |
|  |  | *<50 years,*  *n=10* | *50-65 years.*  *n=25* | *65-75 years,*  *n=17* | *>75 years,*  *n=11* |
| Before RBC transfusion | 55.0  (49.6; 63.0),  51 | 62.5  (57.3; 63.5),  8 | 59.4  (50.0;63.0),  22 | 50.0  (46.7; 59.3),  16 | 56.9  (51.1; 62.8),  9 |
| End of RBC transfusion | 51.0  (41.5; 61.2),  54 | 49.9  (32.8; 55.7),  7 | 52.4  (44.5; 65.7),  23 | 49.8  (26.9; 54.2),  16 | 51.0  (45.0; 62.5),  10 |
| 0.5 hr after RBC transfusion | 49.4  (33.2; 57.7),  55 | 58.9  (51.5; 61.0),  7 | 52.3  (31.4; 59.8),  24 | 42.7  (25.4; 51.2),  16 | 46.7  (43.9; 52.6),  10 |
| 1 hr after RBC transfusion | 47.5  (33.4; 59.4),  55 | 54.0  (50.3; 59.5),  8 | 51.2  (43.5; 61.5),  23 | 34.0  (26.1; 47.5),  15 | 46.3  (33.9; 51.8),  11 |
| 3 hr after RBC transfusion | 45.6  (23.8; 63.6),  50 | 51.7  (37.2; 58.5),  8 | 46.8  (23.8; 77.6),  20 | 31.6  (19.9; 46.6),  14 | 55.3  (35.0; 71.3),  10 |
| 24 hr after RBC transfusion | 67.3  (41.6; 83.7),  44 | 72.3  (43.5; 82.3),  8 | 68.9  (47.2; 85.9),  18 | 73.9  (41.8; 85.5),  11 | 55.0  (41.4; 68.5),  9 |

1 MitoPO_2_ mitochondrial oxygen tension measured with the COMET system

**Supplemental table 10:** Mitochondrial oxygenation tension measured with COMET in critically ill patients receiving RBC transfusion in the total study population and stratified according to the BMI of the patient

| Measurement time | MitoPO_2_^1^ in mmHg in all critically ill patients,  median (IQR), n | MitoPO_2_^1^ in mmHg in strata of critically ill patients with anemia according to BMI of patient,  median (IQR), n | | |
| --- | --- | --- | --- | --- |
|  |  | *18-25 kg/m^2^,*  *n=24* | *25-30 kg/m^2^,*  *n=18* | *>30 kg/m^2^,*  *n=21* |
| Before RBC transfusion | 55.0 (49.6; 63.0),  51 | 62.7 (54.2; 65.7),  20 | 53.9 (45.4; 61.0),  14 | 51.7 (49.3; 62.0),  18 |
| End of RBC transfusion | 51.0 (41.5; 61.2),  54 | 53.5 (37.8; 62.7),  23 | 50.5 (42.3; 55.8),  15 | 50.9 (43.7; 54.1),  17 |
| 0.5 hr after RBC transfusion | 49.4 (33.2; 57.7),  55 | 52.4 (35.3; 59.4),  23 | 50.5 (31.5; 57.5),  16 | 46.6 (33.5; 52.0),  17 |
| 1 hr after RBC transfusion | 47.5 (33.4; 59.4),  55 | 50.8 (31.1; 59.5),  22 | 47.5 (27.7; 56.5),  18 | 47.1 (42.5; 58.1),  17 |
| 3 hr after RBC transfusion | 45.6 (23.8; 63.6),  50 | 48.9 (25.2; 65.1),  22 | 35.1 (16.8; 55.3),  14 | 47.8 (31.7; 77.5),  15 |
| 24 hr after RBC transfusion | 67.3 (41.6; 83.7),  44 | 76.2 (54.5; 91.3),  24 | 71.3 (31.5; 79.6),  11 | 43.5 (28.6; 63.9),  11 |

*BMI* Body mass index; *MitoPO_2_* mitochondrial oxygen tension measured with the COMET system

1 MitoPO_2_ mitochondrial oxygen tension measured with the COMET system

**Supplemental table 11:** Mitochondrial oxygenation tension measured with COMET in critically ill patients receiving RBC transfusion in the total study population and stratified according to the APACHE IV score of the patient

| Measurement time | MitoPO_2_^1^ in mmHg in all critically ill patients,  median (IQR), n | MitoPO_2_^1^ in mmHg in strata of critically ill patients with anemia according to APACHE IV score of patient,  median (IQR), n | | |
| --- | --- | --- | --- | --- |
|  |  | *<50,*  *n=8* | *50-80,*  *n=29* | *>80,*  *n=24* |
| Before RBC transfusion | 55.0 (49.6; 63.0),  51 | 61.1 (59.0; 83.9),  7 | 53.1 (46.3; 62.5),  27 | 55.0 (50.5; 63.6),  17 |
| End of RBC transfusion | 51.0 (41.5; 61.2),  54 | 53.5 (49.4; 62.2),  8 | 53.0 (48.6; 57.4),  24 | 45.0 (17.8; 63.0),  21 |
| 0.5 hr after RBC transfusion | 49.4 (33.2; 57.7),  55 | 41.4 (32.0; 56.3),  8 | 49.4 (36.5; 55.7),  24 | 48.7 (28.7; 58.3),  23 |
| 1 hr after RBC transfusion | 47.5 (33.4; 59.4),  55 | 45.1 (41.9; 55.6),  8 | 48.2 (32.4; 54.4),  25 | 47.5 (29.9; 60.1),  21 |
| 3 hr after RBC transfusion | 45.6 (23.8; 63.6),  50 | 46.8 (43.7; 66.6),  8 | 41.3 (26.1; 56.2),  23 | 40.3 (20.6; 63.5),  19 |
| 24 hr after RBC transfusion | 67.3 (41.6; 83.7),  44 | 63.5 (52.4; 86.5),  8 | 61.0 (38.1; 74.1),  21 | 80.6 (33.0; 93.0),  15 |

*APACHE* Acute Physiology and Chronic Health Evaluation; *MitoPO_2_* mitochondrial oxygen tension measured with the COMET system

1 MitoPO_2_ mitochondrial oxygen tension measured with the COMET system

**Supplemental table 12:** Mitochondrial oxygenation tension measured with COMET in critically ill patients receiving RBC transfusion in the total study population and stratified according to the reason of admission to the ICU

| Measurement time | MitoPO_2_^1^ in mmHg in all critically ill patients,  median (IQR), n | MitoPO_2_^1^ in mmHg in strata of critically ill patients with anemia according to reason of admission to ICU,  median (IQR), n | |
| --- | --- | --- | --- |
|  |  | *Surgical,*  *n=31* | *Medical.*  *n=32* |
| Before RBC transfusion | 55.0 (49.6; 63.0),  51 | 55.0 (46.0; 63.6),  25 | 59.5 (50.1; 62.9),  24 |
| End of RBC transfusion | 51.0 (41.5; 61.2),  54 | 51.8 (33.5; 59.7),  25 | 50.9 (45.0; 62.6),  29 |
| 0.5 hr after RBC transfusion | 49.4 (33.2; 57.7),  55 | 43.9 (29.9; 54.9),  26 | 51.5 (37.6; 58.0),  29 |
| 1 hr after RBC transfusion | 47.5 (33.4; 59.4),  55 | 42.3 (26.3; 53.6),  26 | 50.8 (44.0; 59.5),  28 |
| 3 hr after RBC transfusion | 45.6 (23.8; 63.6),  50 | 41.3 (17.7; 58.0),  27 | 49.8 (32.5; 70.4),  23 |
| 24 hr after RBC transfusion | 67.3 (41.6; 83.7),  44 | 69.6 (44.2; 83.6),  21 | 65.8 (41.6; 84.3),  24 |

*ICU* Intensive care unit; *MitoPO_2_* mitochondrial oxygen tension measured with the COMET system

1 MitoPO_2_ mitochondrial oxygen tension measured with the COMET system

**Supplemental table 13:** Mitochondrial oxygenation tension measured with COMET in critically ill patients receiving RBC transfusion in the total study population and stratified according to the days admitted to the ICI before inclusion of the patient

| Measurement time | MitoPO_2_^1^ in mmHg in all critically ill patients,  median (IQR), n | MitoPO_2_^1^ in mmHg in strata of critically ill patients with anemia according to days admitted to ICU at inclusion,  median (IQR), n | |
| --- | --- | --- | --- |
|  |  | *<6 days,*  *n=29* | *≥6 days.*  *n=34* |
| Before RBC transfusion | 55.0 (49.6; 63.0),  51 | 52.1 (47.7; 62.9),  21 | 59.5 (50.5; 63.0),  30 |
| End of RBC transfusion | 51.0 (41.5; 61.2),  54 | 50.7 (43.7; 64.6),  25 | 53.0 (39.7; 59.0),  29 |
| 0.5hr after RBC transfusion | 49.4 (33.2; 57.7),  55 | 45.1 (32.9; 59.6),  25 | 51.5 (39.4; 56.3),  30 |
| 1 hr after RBC transfusion | 47.5 (33.4; 59.4),  55 | 47.2 (38.9; 56.7),  25 | 48.2 (30.0; 61.8),  31 |
| 3 hr after RBC transfusion | 45.6 (23.8; 63.6),  50 | 46.8 (35.0; 66.1),  22 | 42.3 (23.2; 57.5),  28 |
| 24 hr after RBC transfusion | 67.3 (41.6; 83.7),  44 | 57.2 (38.0; 75.2),  19 | 72.0 (43.5; 87.4),  25 |

*ICU* Intensive care unit; *MitoPO_2_* mitochondrial oxygen tension measured with the COMET system

1 MitoPO_2_ mitochondrial oxygen tension measured with the COMET system

**Supplemental table 14:** Mitochondrial oxygenation tension measured with COMET in critically ill patients receiving RBC transfusion in the total study population and stratified according to the total ICU admission duration of the patient

| Measurement time | MitoPO_2_^1^ in mmHg in all critically ill patients,  median (IQR), n | MitoPO_2_^1^ in mmHg in strata of critically ill patients with anemia according to total ICU admission duration,  median (IQR), n | | | |
| --- | --- | --- | --- | --- | --- |
|  |  | *<5 days,*  *n=8* | *5-10 days.*  *n=13* | *11-20 days,*  *n=15* | *>20 days,*  *n=27* |
| Before RBC transfusion | 55.0 (49.6; 63.0),  51 | 50.0  (43.1; 59.0),  8 | 49.4  (45.3;60.2),  9 | 53.5  (50.8; 61.2),  15 | 61.4  (50.3; 63.6),  22 |
| End of RBC transfusion | 51.0 (41.5; 61.2),  54 | 50.7  (42.0; 61.3),  7 | 51.0  (45.0; 55.0),  10 | 50.9  (46.0; 66.3),  14 | 51.7  (33.5; 56.9),  25 |
| 0.5hr after RBC transfusion | 49.4 (33.2; 57.7),  55 | 52.9  (37.2; 64.5),  7 | 42.7  (30.7; 58.0),  11 | 47.8  (39.4; 54.9),  14 | 51.6  (30.6; 56.1),  25 |
| 1 hr after RBC transfusion | 47.5 (33.4; 59.4),  55 | 53.5  (43.3; 63.1),  5 | 40.4  (27.1; 54.4),  13 | 47.4  (44.0; 59.9),  13 | 49.0  (31.1; 59.1),  26 |
| 3 hr after RBC transfusion | 45.6 (23.8; 63.6),  50 | 62.4  (52.8; 66.5),  5 | 42.6  (22.6; 51.7),  13 | 43.4  (27.6; 57.8),  13 | 46.8  (20.1; 61.7),  20 |
| 24 hr after RBC transfusion | 67.3 (41.6; 83.7),  44 | 68.9  (64.5; 79.0),  5 | 49.0  (32.8; 84.0),  9 | 76.2  (33.3; 79.4),  10 | 64.6  (46.7; 83.9),  22 |

*ICU* Intensive care unit; *MitoPO_2_* mitochondrial oxygen tension measured with the COMET system

1 MitoPO_2_ mitochondrial oxygen tension measured with the COMET system

**Supplemental table 15:** Mitochondrial oxygenation tension measured with COMET in critically ill patients receiving RBC transfusion in the total study population and stratified according to the hospital admission duration of the patient

| Measurement time | MitoPO_2_^1^ in mmHg in strata of critically ill patients with anemia according to total hospital admission duration, median (IQR), n | | | | |
| --- | --- | --- | --- | --- | --- |
|  | *<10 days,*  *n=6* | *10-20 days,*  *n=12* | *21-30 days.*  *n=11* | *31-50 days,*  *n=14* | *>50 days,*  *n=20* |
| Before RBC transfusion | 53.2  (45.9; 58.3),  6 | 50.8  (43.4; 60.0),  11 | 59.9  (50.0; 63.0),  10 | 59.2  (49.3; 63.8),  11 | 59.1  (51.6; 63.7),  17 |
| End of RBC transfusion | 53.0  (45.0; 64.0),  6 | 46.0  (42.0; 50.9),  10 | 63.0  (51.1; 66.7),  9 | 48.0  (33.9; 56.1),  13 | 52.2  (48.7; 57.8),  19 |
| 0.5hr after RBC transfusion | 59.0  (46.4; 63.1),  6 | 36.1  (30.4; 47.9),  11 | 53.9  (38.5; 57.3),  9 | 53.8  (45.7; 60.1),  13 | 47.3  (30.2; 52.5),  19 |
| 1 hr after RBC transfusion | 59.5  (57.6; 59.9),  4 | 47.5  (37.9; 52.9),  11 | 48.5  (41.6; 65.3),  9 | 47.2  (31.7; 58.1),  14 | 45.7  (30.0; 58.5),  19 |
| 3 hr after RBC transfusion | 81.1  (65.4; 96.9),  5 | 40.3  (29.7; 46.2),  12 | 52.5  (33.5; 64.3),  9 | 23.7  (20.6; 52.0),  11 | 47.8  (18.9; 69.7),  15 |
| 24 hr after RBC transfusion | 98.6  (82.9; 114.3),  3 | 70.6  (39.7; 94.6),  10 | 65.8  (53.8; 77.2),  10 | 78.1  (31.8; 89.9),  9 | 55.3  (45.0; 76.3),  15 |

1 MitoPO_2_ mitochondrial oxygen tension measured with the COMET system

**Supplemental table 16:** Mitochondrial oxygenation tension measured with COMET in critically ill patients receiving RBC transfusion in the total study population and stratified according to the ICU or in-hospital mortality of the patient

| Measurement time | MitoPO_2_^1^ in mmHg in all critically ill patients,  median (IQR) | MitoPO_2_^1^ in mmHg in strata of critically ill patients with anemia according to ICU of in-hospital mortality,  median (IQR), n | |
| --- | --- | --- | --- |
|  |  | *Not deceased,*  *n=44* | *Deceased.*  *n=19* |
| Before RBC transfusion | 55.0 (49.6; 63.0),  51 | 55.0 (49.1; 62.7),  36 | 58.6 (51.2; 63.6),  15 |
| End of RBC transfusion | 51.0 (41.5; 61.2),  54 | 52.5 (43.2; 63.4),  38 | 46.0 (34.0; 57.4),  16 |
| 0.5hr after RBC transfusion | 49.4 (33.2; 57.7),  55 | 50.0 (33.4; 58.7),  39 | 46.4 (32.7; 56.1),  16 |
| 1 hr after RBC transfusion | 47.5 (33.4; 59.4),  55 | 48.2 (33.4; 60.1),  39 | 47.5 (40.0; 54.4),  16 |
| 3 hr after RBC transfusion | 45.6 (23.8; 63.6),  50 | 45.6 (22.1; 66.5),  33 | 44.6 (24.9; 57.1),  17 |
| 24 hr after RBC transfusion | 67.3 (41.6; 83.7),  44 | 58.2 (33.3; 79.4),  29 | 77.9 (56.8; 93.0),  15 |

*ICU* Intensive care unit; *MitoPO_2_* mitochondrial oxygen tension measured with the COMET system

1 MitoPO_2_ mitochondrial oxygen tension measured with the COMET system

**Supplemental table 17:** Mitochondrial oxygen consumption measured with a linear function on the fitted sigmoid curve of mitoPO_2_ values measured with the COMET in critically ill patients receiving RBC transfusion in the total study population, and stratified according to pre-transfusion hemoglobin concentration

| Measurement time | MitoVO_2_^1^ in mmHg/s in all critically ill patients,  median (IQR), n | MitoVO_2_^1^ in mmHg/s in strata of critically ill patients with anemia according to Hb concentration before RBC transfusion,  median (IQR), n | |
| --- | --- | --- | --- |
|  |  | *Hb concentration ≤ 7 g/dL,*  *n=12* | *Hb concentration >7 g/dL,*  *n=51* |
| Before RBC transfusion | 3.3 (2.1; 5.9),  27 | 7.6 (4.6; 10.5),  3 | 3.3 (2.2; 5.7),  24 |
| End of RBC transfusion | 3.2 (2.1; 5.5),  29 | 5.3 (2.7; 8.3),  8 | 3.1 (2.0; 4.7),  21 |
| 0.5hr after RBC transfusion | 3.7 (2.0; 5.1),  36 | 5.4 (2.4; 8.2),  8 | 3.7 (2.1; 4.5),  28 |
| 1 hr after RBC transfusion | 2.8 (2.2; 4.0),  36 | 3.8 (2.3; 4.5),  6 | 2.7 (2.0; 4.0),  30 |
| 3 hr after RBC transfusion | 2.8 (1.8; 4.2),  28 | 4.2 (3.8; 6.4),  4 | 2.4 (1.8; 4.2),  24 |
| 24 hr after RBC transfusion | 3.1 (2.5; 4.8),  34 | 3.8 (3.3; 3.9),  5 | 3.0 (2.5; 5.0),  29 |

1 MitoVO_2_ mitochondrial oxygen consumption calculated with a linear function on a fitted sigmoid curve of the mitoPO_2_ measurements

**Supplemental table 18**: Overview of the signal quality of mitoPO_2_ measurements with the COMET measurement device over time in all critically ill patients with anemia receiving RBC transfusion, and in strata of participating study centers. An increase in signal quality is seen over time in both participating centers

| Measurement time | Overall signal quality in %,  median (IQR) | Signal quality in % in LUMC, median (IQR) | Signal quality in % in AMC, median (IQR) |
| --- | --- | --- | --- |
| Before RBC transfusion | 32.1 (25.7-38.2) | 34.3 (28.3-39.5) | 26.8 (24.0-31.3) |
| At the end of RBC transfusion | 35.9 (27.4-44.0) | 37.6 (26.7-49.8) | 31.7 (29.9-36.3) |
| 0.5 hour after RBC transfusion | 37.5 (28.0-47.0) | 40.7 (30.1-49.9) | 31.8 (26.9-37.1) |
| 1 hour after RBC transfusion | 40.3 (28.2-55.0) | 44.5 (31.5-57.5) | 29.0 (24.4-35.1) |
| 3 hour after RBC transfusion | 45.9 (30.5-54.6) | 48.6 (36.6-56.9) | 27.8 (23.4-33.0) |
| 24 hour after RBC transfusion | 42.9 (38.7-57.3) | 45.9 (39.4-60.3) | 41.0 (31.2-41.3) |

*AMC* Amsterdam University Medical Center; *LUMC* Leiden University Medical Center; *MitoPO_2_* mitochondrial oxygen tension measured with the COMET system; *RBC* Red blood cell

**Supplemental table 19**: Association between mitoPO_2_ and mitoVO_2_ measurements with markers of tissue perfusion. No statistical different mitoPO_2_ or mitoVO_2_ values were observed between categories of markers of tissue perfusion

| Characteristics | Number of measurements included  (total n=301)^1^ | MitoPO_2_ in mmHg,  median (IQR) | Mean difference (95% CI) | p-value for trend | Number of measurements included  (total n=174)^2^ | MitoVO_2_ in mmHg/s, median (IQR) | Mean difference  (95% CI) | p-value for trend |
| --- | --- | --- | --- | --- | --- | --- | --- | --- |
| MAP  <65 mmHg  65-95 mmHg  >95 mmHg | 18  246  37 | 45.9 (34.0; 51.4)  51.8 (36.5; 63.8)  52.2 (42.3; 58.2) | *reference*  5.4 (-5.9; 16.8)  4.6 (-8.7; 17.9) | 0.634 | 11  143  20 | 2.44 (2.06; 4.80)  3.30 (2.08; 4.80)  3.03 (1.99; 3.80) | *reference*  -1.04 (-2.81; 0.74)  -1.73 (-3.86; 0.40) | 0.278 |
| Vasopressor use  No  Yes  *Missing* | 149  151  *1* | 51.5 (39.0; 67.3)  50.9 (39.5; 62.5)  *54.0 (54.0; 54.0)* | *reference*  -4.0 (-9.3; 1.3) | 0.142 | 86  87  *1* | 3.15 (2.15; 4.88)  3.09 (2.00; 4.53)  *12.20 (12.20; 12.20)* | *reference*  -0.55 (-1.39; 0.30) | 0.202 |
| Lactate  < 2mmol/L  ≥2 mmol/L  *Missing* | 234  46  *21* | 51.3 (39.8; 62.9)  47.1 (26.7; 65.1)  *58.7 (46.0; 72.0)* | *reference*  -0.6 (-8.1; 6.8) | 0.865 | 134  28  *12* | 3.11 (1.99; 4.71)  3.00 (2.21; 4.61)  *3.58 (2.72; 6.21)* | *reference*  0.26 (-0.83; 1.36) | 0.633 |
| Lactate one measurement later  < 2 mmol/L  ≥ 2 mmol/L  *Missing* | 184  33  *84* | 50.6 (38.2; 60.1)  46.0 (23.9; 62.8)  *57.2 (45.9; 79.9)* | *reference*  -2.8 (-10.4; 4.8) | 0.475 | 102  19  *53* | 2.89 (1.98; 4.36)  3.74 (1.57; 5.34)  *3.40 (2.59; 4.68)* | *reference*  0.68 (-0.66; 2.01) | 0.317 |
| ScvO2  < 70%  ≥ 70%  *Missing* | 77  90  *134* | 53.0 (35.1; 63.5)  53.3 (35.0; 63.6)  *49.9 (41.3; 62.7)* | *reference*  0.5 (-7.1; 8.0) | 0.905 | 46  52  *76* | 2.89 (1.79; 4.12)  3.59 (2.31; 4.94)  *3.10 (2.13; 4.75)* | *reference*  0.45 (-0.47; 1.38) | 0.335 |
| pCO2 gap  < 3.5 mmHg  ≥ 3.5 mmHg  *Missing* | 48  89  *164* | 54.8 (34.9; 64.4)  53.5 (35.2; 65.8)  *54.8 (34.9; 64.4)* | *reference*  0.3 (-7.9; 8.6) | 0.935 | 24  50  *100* | 3.41 (2.42; 4.08)  2.99 (1.79; 4.35)  *3.10 (2.13; 5.11)* | *reference*  0.04 (-1.07; 1.15) | 0.944 |
| Cardiac index  < 3.9  ≥ 3.9  *Missing* | 61  52  *188* | 51.2 (35.0; 68.0)  57.4 (39.0; 63.1)  *50.8 (39.8; 62.8)* | *reference*  1.3 (-8.0; 10.7) | 0.776 | 45  28  *101* | 3.15 (2.00; 4.78)  2.37 (1.73; 3.52)  *3.40 (2.32; 4.96)* | *reference*  -0.78 (-1.89; 0.34) | 0.170 |
| Fractional inspired oxygen  ≤ 30%  > 30%  *Missing* | 116  142  *43* | 51.8 (35.1; 63.5)  52.0 (41.0; 62.7)  *47.4 (30.8; 63.5)* | *reference*  1.9 (-3.8; 7.6) | 0.510 | 69  90  *15* | 2.92 (2.01; 4.25)  3.47 (2.30; 4.95)  *3.60 (1.90; 5.86)* | *reference*  0.82 (-0.08; 1.71) | 0.074 |
| Fluid balance  < 0L  0-1L  1-2.5L  > 2.5L  *Missing* | 69  83  67  24  *58* | 50.0 (33.0; 62.5)  50.0 (40.4; 62.5)  55.2 (37.8; 65.2)  37.9 (26.7; 54.2)  *53.8 (47.5; 67.5)* | *reference*  -2.5 (-10.1; 5.2)  2.8 (-5.2; 10.8)  -10.8 (-22.0; 0.3) | 0.104 | 37  61  44  6  *26* | 2.35 (1.68; 3.60)  3.09 (2.22; 4.96)  3.71 (2.29; 4.46)  2.67 (1.78; 3.50)  *3.76 (2.70; 7.33)* | *reference*  2.96 (0.01; 2.18)  1.09 (-0.16; 2.16)  1.00 (-1.28; 3.31) | 0.221 |

*MitoPO_2_* mitochondrial oxygen tension measured with the COMET system; MitoVO_2_ mitochondrial oxygen consumption measured with a linear function on a fitted sigmoid curve; *pCO_2_* *gap* venous-to-arterial carbon dioxide difference; *ScvO_2_* central venous oxygen saturation

^1^ number of mitoPO_2_ measurements

^2^ number of mitoVO_2_ measurements

**Supplemental table 20:** Description of missing mitoPO_2_ measurement with a signal quality of at least 10%, per measurement moment and overall as part of the sensitivity analyses. Most missing at the start of the measurements was due to poor signal quality. Over time, missing is more due to logistical problems

| Measurement time | Missing mitoPO_2_ measurements,  n (% total)^1,2^ | Reason Missing |
| --- | --- | --- |
| Before RBC transfusion | 2 (3.2%) | - Signal quality <20% (n=2) |
| End of RBC transfusion | 5 (7.9%) | - Signal quality <20% (n=2) - Logistical problems (n=2) - Measurement data not saved (n=1) |
| 0.5hour after RBC transfusion | 6 (9.5%) | - Signal quality <20% (n=3) - Measurement data not saved (n=1) - Logistical problems (n=1) - Technical problems measurement device (n=1) |
| 1 hour after RBC transfusion | 7 (11.1%) | - Signal quality <20% (n=3) - Logistical problems (n=2) - Measurement data not saved (n=1) - Technical problems measurement device (n=1) |
| 3 hours after RBC transfusion | 13 (20.6%) | - Logistical problems (n=11) - Measurement data not saved (n=1) - Technical problems measurement device (n=1) |
| 24 hours after RBC transfusion | 20 (31.7%) | - Logistical problems (n=16) - Measurement data not saved (n=2) - Signal quality <20% (n=1) - Technical problems measurement device (n=1) |
| *Overall* | *53 (14.0%)* | - *Logistical problems (n=32)* - *Signal quality <20% (n=11)* - *Measurement data not saved (n=6)* - *Technical problems measurement device(n=4)* |

1 MitoPO_2_ mitochondrial oxygen tension measured with the COMET system

2 There were a total of 63 mitoPO_2_ measurements per measurement time, with in total 378 measurements

**Supplemental table 21**: Overview of demographic characteristics of our study cohort with no missing mitoPO_2_ measurements and the study cohort with missing mitoPO_2_ measurements due to a signal quality of less than 10% as part of the sensitivity analyses. The median BMI, and APACHE IV score were higher in the critically ill patients without valid mitoPO_2_ measurements compared to patients with valid mitoPO_2_ measurements

| Characteristic | Cohort with no missing mitoPO_2_ due to low signal quality (n=54) | Cohort with missing mitoPO_2_ due to signal quality <10% (n=9) |
| --- | --- | --- |
| Age in years, median (IQR) | 64.0 (53.5; 72.8) | 70.0 (64.0; 75.0) |
| Female sex, n (%) | 14 (26%) | 1 (11%) |
| BMI, median (IQR) | 27.3 (23.8; 32.2) | 29.8 (25.1; 33.5) |
| Admission reason, n(%)  Surgical  Non-surgical | 24 (44%)  30 (56%) | 7 (78%)  2 (22%) |
| Chronic comorbidities, n(%)  No known comorbidity  Chronic Cardiovascular Insufficiency  Chronic Obstructive Lung Disease  Chronic Renal Insufficiency  Chronic dialysis  Cirrhosis  Hematologic malignancy  Immunologic deficiency  Diabetes mellitus | 18 (33%)  5 (9%)  1 (2%)  2 (4%)  1 (2%)  5 (9%)  3 (6%)  2 (4%)  17 (31%) | 4 (44%)  0 (0%)  0 (0%)  0 (0%)  0 (0%)  0 (0%)  0 (0%)  0 (0%)  5 (56%) |
| SOFA score before transfusion, median (IQR) | 10.0 (8.3; 11.8) | 10.0 (6.0; 11.0) |
| APACHE IV score, median (IQR) | 70.0 (57.0; 88.0) | 80.0 (63.0; 91.0) |
| Hemoglobin concentration before transfusion in g/dL, median (IQR) | 7.4 (7.1; 7.7) | 7.6 (7.3; 7.7.9) |
| Hematocrit before transfusion in L/L, median (IQR) | 0.23 (0.22; 0.24) | 0.23 (0.22; 0.24) |
| Days admitted to ICU at inclusion, median (IQR) | 6.0 (3.0; 11.8) | 6.0 (3.0; 10.0) |
| Total ICU admission duration in days, median (IQR) | 18.0 (9.3; 31.5) | 11.0 (8.0; 24.0) |
| Total hospital admission duration in days, median (IQR) | 33.5 (18.5; 59.8) | 31.0 (23.0; 33.0) |
| ICU or in-hospital mortality, n(%) | 16 (30%) | 3 (33%) |

*APACHE* Acute Physiology and Chronic Health Evaluation; *BMI* Body mass index*; CI* confidence interval; *ICU* Intensive care unit; *SOFA* Sequential Organ Failure Assessment

**Supplemental table 22:** Mitochondrial oxygenation tension with a signal quality of at least 10% in critically ill patients receiving RBC transfusion in the total study population and stratified according to pre-transfusion hemoglobin concentration as part of the sensitivity analyses

| Measurement time | MitoPO_2_^1^ in mmHg in all critically ill patients,  median (IQR) - n | MitoPO_2_^1^ in mmHg in strata of critically ill patients with anemia based on Hb concentration before RBC transfusion,  median (IQR) - n | |
| --- | --- | --- | --- |
|  |  | *Hb concentration ≤ 7 g/dL,*  *n=12* | *Hb concentration >7 g/dL,*  *n=51* |
| Before RBC transfusion | 54.1 (49.1; 62.1) -  60 | 57.3 (48.7; 62.0) -  12 | 54.1 (49.0; 62.1) -  49 |
| End of RBC transfusion | 51.0 (39.6; 60.5) -  59 | 47.2 (30.1; 56.4) -  12 | 51.2 (40.3; 60.5) -  47 |
| 0.5hr after RBC transfusion | 49.2 (34.3; 57.0) -  57 | 49.9 (36.0; 67.0) -  12 | 49.2 (34.3; 56.3) -  45 |
| 1 hr after RBC transfusion | 47.1 (30.6; 59.5) -  57 | 53.2 (37.0; 74.6) -  12 | 45.6 (30.6; 55.4) -  45 |
| 3 hr after RBC transfusion | 43.7 (24.1; 62.4) -  51 | 49.7 (23.5; 89.6) -  10 | 42.6 (24.8; 58.3) -  42 |
| 24 hr after RBC transfusion | 65.9 (42.6; 84.5) -  43 | 42.6 (25.6; 58.5) -  6 | 68.2 (44.6; 85.2) -  38 |

1 MitoPO_2_ mitochondrial oxygen tension measured with the COMET system

**Supplemental table 23**: Association of mitoPO_2_ (with a signal quality of at least 10%) and mitoVO_2_ values with demographic and outcome characteristics of the study population as part of the sensitivity analyses

| Characteristics | Number of participants with mitoPO_2_ measurement (total 60)^1^ | MitoPO_2_ in mmHg, median (IQR) | Mean difference (95% CI) | p-value for trend | Number of participants with mitoVO_2_ measurement (total 26)^2^ | MitoVO_2_ in mmHg/s, median (IQR) | Mean difference (95% CI) | p-value for trend |
| --- | --- | --- | --- | --- | --- | --- | --- | --- |
| **Demographic characteristics** |  | | | |  | | |  |
| Sex  Female  Male | 15  45 | 52.9 (48.7; 61.5)  54.6 (49.1; 62.3) | *ref*  -1.9 (-10.9; 7.0) | 0.671 | 4  22 | 1.99 (1.60; 3.00)  3.70 (2.34; 7.03) | *reference*  2.56 (-1.53; 6.65) | 0.210 |
| Age  < 50 years  50-64 years  65-75 years  >75 years | 10  23  17  10 | 60.0 (50.8; 63.7)  57.9 (51.8; 62.1)  49.1 (42.1; 56.8)  57.2 (53.0; 61.4) | *ref*  -0.1 (-11.4; 11.1)  -8.0 (-19.9; 3.9)  -3.8 (-17.1; 9.6) | 0.374 | 4  11  8  3 | 2.83 (1.92;4.94)  3.87 (2.23; 6.70)  3.98 (2.54; 7.13)  2.88 (1.93; 3.40) | *reference*  0.97 (-3.64; 5.58)  1.58 (-3.26; 6.42)  -1.44 (-7.47; 4.60) | 0.673 |
| BMI  <25 kg/m^2^  25-30 kg/m^2^  >30 kg/m^2^ | 22  17  21 | 60.8 (55.2; 65.6)  53.3 (45.0; 60.8)  50.3 (49.1; 61.2) | *ref*  -8.8 (-18.3; 0.7)  -6.2 (-15.1; 2.8) | 0.161 | 9  7  10 | 3.91 (2.12; 5.09)  3.09 (2.85; 4.74)  2.92 (1.81; 10.32) | *reference*  -0.51 (-4.43; 3.40)  1.41 (-2.16; 4.98) | 0.547 |
| APACHE IV scoreᶧ  <50  50-80  >80  *missing* | 7  28  23  *2* | 59.5 (56.7; 72.5)  53.4 (48.1; 62.1)  52.9 (49.6; 61.1)  *55.6 (53.0; 58.2)* | *ref*  -9.8 (-22.5; 2.9)  -11.1 (-24.1; 1.8) | 0.223 | 3  14  8  *1* | 10.70 (7.81; 11.25)  2.88 (2.21; 3.90)  3.39 (1.59; 6.36)  *3.53 (3.53; 3.53)* | *reference*  -4.91 (-9.59; -0.24)  -4.92 (-9.90; 0.06) | 0.102 |
| Reason of admission to ICU  Medical  Surgical | 32  28 | 54.1 (50.0; 61.6)  54.5 (44.6; 63.2) | *ref*  -3.2 (-10.9; 4.6) | 0.415 | 10  16 | 2.24 (1.80; 4.67)  3.70 (2.87; 7.20) | *reference*  1.82 (-1.23; 4.86) | 0.230 |
| Hemoglobin concentration before RBC transfusion  < 7 g/dL  ≥ 7 g/dL | 12  48 | 57.3 (48.7; 62.0)  54.1 (49.0; 62.1) | *ref*  -2.0 (-11.7; 7.7) | 0.677 | 2  24 | 7.59 (4.64; 10.55)  3.31 (2.16; 5.72) | *reference*  -3.06 (-8.65; 2.52) | 0.269 |
| Days admitted to ICU before inclusion  < 6  ≥ 6 | 27  33 | 52.9 (48.3; 62.1)  58.8 (49.9; 62.0) | *ref*  2.8 (-5.0; 10.5) | 0.479 | 12  14 | 2.98 (1.55; 5.99)  3.89 (2.43; 5.92) | *reference*  0.64 (-2.42; 3.69) | 0.670 |
| **Outcome characteristics** |  | | | |  | | | |
| SOFA score before RBC transfusion  <10  ≥10 | 21  39 | 49.9 (42.1; 60.8)  57.9 (51.9; 62.7) | ref  7.4 (-0.5; 15.3) | 0.067 | 9  17 | 5.61 (1.69; 10.70)  3.09 (2.30; 4.93) | *reference*  -1.65 (-4.78; 1.49) | 0.289 |
| SOFA score change in 24hr  ≥0 (decrease in SOFA score)  <0 (increase in SOFA score)  *missing* | 33  19  *8* | 59.5 (50.3; 63.8)  55.6 (41.6; 61.6)  *51.8 (48.2; 53.5)* | *ref*  7.7 (-16.7; 1.3) | 0.090 | 13  11  *2* | 3.53 (2.18; 6.03)  2.88 (1.90; 5.35)  *7.32 (5.07; 9.56)* | *reference*  0.30 (-2.78; 3.37) | 0.842 |
| Total ICU admission duration  < 5 days  5-10 days  11-20 days  >20 days | 8  11  15  26 | 55.2 (43.2; 62.1)  52.0 (48.1; 55.6)  53.8 (51.0; 61.4)  60.0 (49.6; 63.1) | *ref*  -2.2 (-16.2; 11.9)  4.8 (-8.5; 18.0)  1.6 (-10.6; 13.9) | 0.701 | 2  6  5  13 | 6.56 (3.94; 9.18)  4.35 (2.90; 8.29)  2.88 (2.30; 4.93)  3.53 (2.12; 5.09) | *reference*  -1.10 (-7.65; 5.44)  -1.86 (-8.57; 4.85)  -2.36 (-8.45; 3.73) | 0.824 |
| Total hospital admission duration  < 10 days  10-20 days  21-30 days  31-50 days  >50 days | 6  11  11  13  19 | 55.4 (45.9; 59.8)  52.0 (49.0; 57.8)  60.5 (44.7; 62.0)  55.6 (49.1; 63.4)  54.6 (50.3; 62.7) | *ref*  -0.5 (-16.1; 15.1)  3.0 (-12.6; 18.6)  3.1 (-12.0; 18.3)  2.1 (-12.3; 16.5) | 0.972 | 2  6  5  4  9 | 6.56 (3.94; 9.18)  4.35 (2.50; 8.29)  2.18 (1.69; 4.93)  2.85 (2.65; 4.00)  3.87 (2.88; 5.09) | *reference*  -1.19 (-7.91; 5.52)  -2.26 (-9.13; 4.62)  -2.76 (-9.89; 4.36)  -1.90 (-8.33; 4.53) | 0.926 |
| In-hospital or ICU mortality  No  Yes | 42  18 | 54.1 (48.5; 62.3)  56.6 (50.4; 61.8) | *ref*  1.4 (-7.1; 9.8) | 0.749 | 16  10 | 3.31 (2.01; 5.33)  3.90 (2.21; 6.92) | *reference*  0.14 (-3.00; 3.29) | 0.925 |

*APACHE* Acute Physiology and Chronic Health Evaluation; *BMI* Body mass index*; CI* confidence interval; Hb Hemoglobin; *ICU* Intensive care unit; *MitoPO_2_* mitochondrial oxygen tension measured with the COMET system; MitoVO_2_ mitochondrial oxygen consumption measured with a linear function on a fitted sigmoid curve

^1^ number of participants with mitoPO_2_ measurements before RBC transfusion

^2^ number of participants with mitoVO_2_ measurements before RBC transfusion

**Supplemental table 24**: Characteristics of clinically used surrogate markers of tissue perfusion and oxygenation in critically ill patients in ICU within subgroups of mitoPO_2_ values of <40, 40-70, and >70 mmHg. As part of the sensitivity analyses, mitoPO_2_ measurements with a signal quality of at least 10% were included

| Clinical characteristics | | MitoPO_2_ | | |
| --- | --- | --- | --- | --- |
|  | **<40 mmHg (n=90)** | | **40-70 mmHg (n=183)** | **>70 mmHg (n=51)** |
| MitoPO_2_ in mmHg, median (IQR) | 24.4 (15.6; 32.5) | | 53.0 (48.3; 60.5) | 85.8 (75.1; 92.8) |
| MitoVO_2_ in mmHg/s, median (IQR)  *Missing, n(%)* | 2.44 (1.54; 3.75)  *26 (32.9%)* | | 3.15 (2.20; 4.91)  *86 (50.9%)* | 4.13 (3.00; 5.36)  *15 (28.3%)* |
| MAP in mmHg, median (IQR) | 77.7 (68.8; 86.9) | | 80.0 (72.8; 88.3) | 76.7 (70.8; 87.5) |
| Vasopressor use, n (%)  *Missing, n(%)* | 45 (50%)  *0 (0.0%)* | | 100 (55%)  *1 (0.5%)* | 19 (37%)  *0 (0.0%)* |
| Lactate in mmol/L, median (IQR)  *Missing, n(%)* | 1.5 (1.2; 1.9)  *4 (4.4%)* | | 1.4 (1.0; 1.7)  *14 (7.7%)* | 1.5 (1.2; 1.9)  *5 (9.8%)* |
| Lactate one measurement later in mmol/L, median (IQR)  *Missing, n(%)* | 1.5 (1.3; 1.9)  *18 (20.0%)* | | 1.4 (1.1; 1.7)  *38 (20.8%)* | 1.4 (1.0; 1.8)  *30 (58.8%)* |
| ScvO_2_ in %, median (IQR)  *Missing, n(%)* | 70.0 (64.0.0; 74.0)  *40 (44.4%)* | | 71.0 (63.3; 77.0)  *85 (46.4%)* | 69.4 (59.0; 81.0)  *22 (43.1%)* |
| pCO_2_ gap in mmHg, median (IQR)  *Missing, n(%)* | 4.1 (2.3; 5.4)  *50 (55.6%)* | | 4.5 (2.3; 6.8)  *102 (55.7%)* | 4.1 (2.9; 6.0)  *27 (52.9%)* |
| Cardiac index in L/min/m^2^, median (IQR)  *Missing, n(%)* | 3.8 (3.3; 4.2)  *56 (62.2%)* | | 3.8 (3.0; 5.2)  *122 (66.7%)* | 3.6 (3.1; 4.2)  *31 (60.8%)* |
| Fractional inspired oxygen in %, median (IQR)  *Missing, n(%)* | 35.0 (30.0; 44.0)  *21 (23.3%)* | | 35.0 (29.5; 45.0)  *23 (12.6%)* | 35.0 (25.0; 40.0)  *8 (15.7%)* |
| Fluid balance in L, median (IQR)  *Missing, n(%)* | 0.749 (-0.261; 1.913)  *12 (13.3%)* | | 0.695 (-0.066; 1.412)  *43 (23.5%)* | 0.582 (-0.617; 1.080)  *14 (27.5%)* |

*MitoPO_2_* mitochondrial oxygen tension measured with the COMET system; *pCO_2_* *gap* venous-to-arterial carbon dioxide difference; *ScvO_2_* central venous oxygen saturation; *SOFA* Sequential Organ Function Assessment

**Supplemental table 25**: Association between mitoPO_2_ (with a signal quality of at least 10%) and mitoVO_2_ measurements with markers of tissue perfusion as part of the sensitivity analyses. No statistical different mitoPO_2_ or mitoVO_2_ values were observed between categories of markers of tissue perfusion

| Characteristics | Number of measurements included  (total n=312)^1^ | MitoPO_2_ in mmHg,  median (IQR) | Mean difference (95% CI) | p-value for trend | Number of measurements included  (total n=174)^2^ | MitoVO_2_ in mmHg/s, median (IQR) | Mean difference  (95% CI) | p-value for trend |
| --- | --- | --- | --- | --- | --- | --- | --- | --- |
| MAP  <65 mmHg  65-95 mmHg  >95 mmHg  *Missing* | 21  254  38  *1* | 41.6 (33.6; 51.5)  51.2 (35.3; 63.7)  51.8 (41.6; 61.9)  *57.0 (57.0; 57.0)* | *reference*  6.1 (-4.1; 16.4)  6.7 (-5.6; 19.0) | 0.483 | 11  143  20 | 2.44 (2.06; 4.80)  3.30 (2.08; 4.80)  3.03 (1.99; 3.80) | *reference*  -1.04 (-2.81; 0.74)  -1.73 (-3.86; 0.40) | 0.278 |
| Vasopressor use  No  Yes  *Missing* | 152  159  *1* | 51.4 (35.3; 64.4)  50.6 (37.0; 61.6)  *50.5 (50.5; 50.5)* | *reference*  -2.7 (-7.8; 2.2) |  | 86  87  *1* | 3.15 (2.15; 4.88)  3.09 (2.00; 4.53)  *12.20 (12.20; 12.20)* | *reference*  -0.55 (-1.39; 0.30) | 0.202 |
| Lactate  < 2mmol/L  ≥2 mmol/L  *Missing* | 238  51  *23* | 51.0 (39.4; 61.5)  46.3 (28.9; 65.2)  *58.7 (46.3; 67.5)* | *reference*  -0.1 (-7.1; 6.8) | 0.971 | 134  28  *12* | 3.11 (1.99; 4.71)  3.00 (2.21; 4.61)  *3.58 (2.72; 6.21)* | *reference*  0.26 (-0.83; 1.36) | 0.633 |
| Lactate one measurement later  < 2 mmol/L  ≥ 2 mmol/L  *Missing* | 191  29  *84* | 50.5 (37.7; 60.1)  41.6 (26.7; 61.5)  *58.1 (43.0; 76.4)* | *reference*  -3.1 (-10.0; 3.8) | 0.372 | 102  19  *53* | 2.89 (1.98; 4.36)  3.74 (1.57; 5.34)  *3.40 (2.59; 4.68)* | *reference*  0.68 (-0.66; 2.01) | 0.317 |
| ScvO2  < 70%  ≥ 70%  *Missing* | 81  95  *139* | 53.1 (33.4; 63.7)  52.9 (35.2; 64.1)  *49.8 (39.4; 60.4)* | *reference*  0.7 (-6.4; 7.8) | 0.842 | 46  52  *76* | 2.89 (1.79; 4.12)  3.59 (2.31; 4.94)  *3.10 (2.13; 4.75)* | *reference*  0.45 (-0.47; 1.38) | 0.335 |
| pCO2 gap  < 3.5 mmHg  ≥ 3.5 mmHg  *Missing* | 53  92  *169* | 53.0 (33.5; 63.8)  54.2 (38.6; 66.4)  *49.6 (37.7; 60.4)* | *reference*  2.2 (-5.5; 10.0) | 0.569 | 24  50  *100* | 3.41 (2.42; 4.08)  2.99 (1.79; 4.35)  *3.10 (2.13; 5.11)* | *reference*  0.04 (-1.07; 1.15) | 0.944 |
| Cardiac index  < 3.9  ≥ 3.9  *Missing* | 63  52  *198* | 51.4 (34.7; 67.0)  58.1 (35.2; 65.1)  *50.4 (39.3; 61.1)* | *reference*  2.0 (-7.0; 11.0) | 0.659 | 45  28  *101* | 3.15 (2.00; 4.78)  2.37 (1.73; 3.52)  *3.40 (2.32; 4.96)* | *reference*  -0.78 (-1.89; 0.34) | 0.170 |
| Fractional inspired oxygen  ≤ 30%  > 30%  *Missing* | 116  146  *51* | 52.6 (35.5; 63.7)  51.5 (40.4; 61.5)  *46.6 (24.3; 61.8)* | *reference*  1.5 (-3.9; 7.0) | 0.573 | 69  90  *15* | 2.92 (2.01; 4.25)  3.47 (2.30; 4.95)  *3.60 (1.90; 5.86)* | *reference*  0.82 (-0.08; 1.71) | 0.074 |
| Fluid balance  < 0L  0-1L  1-2.5L  > 2.5L  *Missing* | 73  86  66  24  *67* | 48.5 (29.8; 60.8)  49.6 (36.0; 61.1)  55.7 (40.1; 65.4)  37.7 (28.0; 53.2)  *53.4 (46.9; 63.5)* | *reference*  -1.0 (-8.2; 6.1)  4.8 (-2.9; 12.4)  -8.6 (-19.2; 2.1) | 0.096 | 37  61  44  6  *26* | 2.35 (1.68; 3.60)  3.09 (2.22; 4.96)  3.71 (2.29; 4.46)  2.67 (1.78; 3.50)  *3.76 (2.70; 7.33)* | *reference*  2.96 (0.01; 2.18)  1.09 (-0.16; 2.16)  1.00 (-1.28; 3.31) | 0.221 |

*MitoPO_2_* mitochondrial oxygen tension measured with the COMET system; MitoVO_2_ mitochondrial oxygen consumption measured with a linear function on a fitted sigmoid curve; *pCO_2_* *gap* venous-to-arterial carbon dioxide difference; *ScvO_2_* central venous oxygen saturation

^1^ number of mitoPO_2_ measurements

^2^ number of mitoVO_2_ measurements
